# A Computational and Experimental Approach Linking Disorder, High‐Pressure Behavior, and Mechanical Properties in UiO Frameworks

**DOI:** 10.1002/anie.201509352

**Published:** 2016-01-21

**Authors:** Claire L. Hobday, Ross J. Marshall, Colin F. Murphie, Jorge Sotelo, Tom Richards, David R. Allan, Tina Düren, François‐Xavier Coudert, Ross S. Forgan, Carole A. Morrison, Stephen A. Moggach, Thomas D. Bennett

**Affiliations:** ^1^ EaStCHEM School of Chemistry and Centre for Science at Extreme ConditionsUniversity of EdinburghDavid Brewster Road, Joseph Black BuildingEdinburghEH9 3FJUK; ^2^ WestCHEMSchool of ChemistryThe University of GlasgowUniversity AvenueGlasgowG12 8QQUK; ^3^ Department of Materials Science and MetallurgyUniversity of CambridgeCharles Babbage RoadCambridgeCB3 0FSUK; ^4^ Diamond Light SourceHarwell CampusDidcotOX11 ODEUK; ^5^ Department of Chemical EngineeringUniversity of BathClaverton DownBathBA2 7AYUK; ^6^ Chimie ParisTechPSL Research University, CNRS, Institut de Recherche de Chimie75005ParisFrance

**Keywords:** gas separation, high-pressure chemistry, metal–organic frameworks, structure elucidation, X-ray crystallography

## Abstract

Whilst many metal–organic frameworks possess the chemical stability needed to be used as functional materials, they often lack the physical strength required for industrial applications. Herein, we have investigated the mechanical properties of two UiO‐topology Zr‐MOFs, the planar UiO‐67 ([Zr_6_O_4_(OH)_4_(bpdc)_6_], bpdc: 4,4′‐biphenyl dicarboxylate) and UiO‐abdc ([Zr_6_O_4_(OH)_4_(abdc)_6_], abdc: 4,4′‐azobenzene dicarboxylate) by single‐crystal nanoindentation, high‐pressure X‐ray diffraction, density functional theory calculations, and first‐principles molecular dynamics. On increasing pressure, both UiO‐67 and UiO‐abdc were found to be incompressible when filled with methanol molecules within a diamond anvil cell. Stabilization in both cases is attributed to dynamical linker disorder. The diazo‐linker of UiO‐abdc possesses local site disorder, which, in conjunction with its longer nature, also decreases the capacity of the framework to compress and stabilizes it against direct compression, compared to UiO‐67, characterized by a large elastic modulus. The use of non‐linear linkers in the synthesis of UiO‐MOFs therefore creates MOFs that have more rigid mechanical properties over a larger pressure range.

Metal–organic frameworks (MOFs) continue to be of exceptional interest to the scientific community because of their guest‐specific gas sorption, separation, drug‐delivery, and catalytic properties.^1^ Whilst significant progress has been made in increasing the chemical stability of MOFs,^2^ their “soft” mechanical properties often lead to framework collapse or structural distortion upon application of temperature, shear stress, or hydrostatic pressure.^3^ This poses a severe problem during sintering and pelletization processes, used to shape MOF powders into industrially useful morphologies.^4^ Any structural deformation however also alters the highly selective guest‐binding properties of MOFs, which therefore means that structural durability is a highly desired quality. Routes to such mechanically robust structures have included embedding MOFs into polymer matrices, or coating nanoparticles with silica,^5^ although both lead to substantial deterioration in guest sorption ability.

While sometimes problematic for applications, structural flexibility does however give rise to a very rich and diverse array of pressure‐ and temperature‐induced mechanical responses in MOFs, which may be tuned to individual application needs.^6^ The importance of this mechanical behavior motivated us to investigate the link between stimuli‐induced mechanical response and chemical structure in the well‐known UiO family of Zr‐MOFs.

The isoreticular series of UiO‐type MOFs consists of Zr_6_O_4_(OH)_4_ nodes, which are interconnected by linear or bent dicarboxylate ligands.^7^ The high valency of Zr^IV^ and 12‐fold coordination of the metal cluster are associated with high shear and bulk moduli, which surpass those of other MOFs.^8^ In this work, we build upon recent advances in the synthesis of single crystals of UiO frameworks, and present a combined computational and experimental study of the mechanical behavior of UiO‐67 [Zr_6_O_4_(OH)_4_(bpdc)_6_] (bpdc: 4,4′‐biphenyl dicarboxylate)^9^ and an azobenzene derivative, UiO‐abdc [Zr_6_O_4_(OH)_4_(abdc)_6_] (abdc: 4,4′‐azobenzene dicarboxylate)^10^ (Figure 1). A combination of single‐crystal nanoindentation, high‐pressure X‐ray diffraction studies, density functional theory (DFT) calculations, and first‐principles molecular dynamics (MD) simulations were used to show that the dynamic disorder induced in UiO‐abdc by the ligand has a significant impact upon the mechanical behavior of the framework.

**Figure 1:**
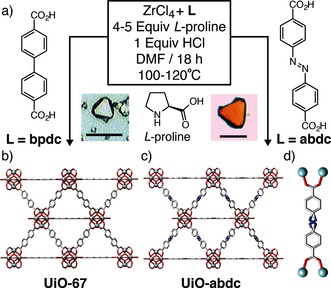
a) Synthetic, modulator‐based pathway to UiO‐67 and UiO‐abdc. b) Unit cell of UiO‐67. c) Unit cell of UiO‐abdc. d) Enlarged abdc linker. C: grey, O: red, Zr: light blue, N: dark blue, H omitted for clarity. Scale bar=100 μm.

Synthetic conditions leading to single‐crystals of UiO‐type MOFs are rare and typically require the addition of a significant excess of monocarboxylic acid crystallization modulators.^11^ We have found the amino acid l‐proline to be a highly efficient modulator in the synthesis of Zr UiO‐MOFs.^12^ Addition of 5 and 1 equivalents (with respect to the linker) of l‐proline and HCl (a known synthetic promoter^13^) during solvothermal syntheses yielded single crystals of ≈50 μm diameter of both UiO‐67 and UiO‐abdc (Supporting Information, Section SI‐1). Both UiO‐67 and UiO‐abdc crystallize in the cubic space group $Fm\bar{3}m$ (*a*≈26.85 Å and 29.32 Å respectively), and contain octahedral cages of diameter 16 Å (UiO‐67) and 19 Å (UiO‐abdc), which share faces with eight smaller tetrahedral pores of diameters 12 Å (UiO‐67) and 15 Å (UiO‐abdc).^10^

Room temperature single‐crystal diffraction data were collected to compare to our high‐pressure data at room temperature. For UiO‐67, some libration was observed on the bpdc ligand, while much larger ellipsoids and more disorder were apparent in the abdc ligand in UiO‐abdc. Phenyl rings in UiO‐abdc were modelled over three positions (one half‐occupied, the other quarter‐occupied over two positions), while the diazo moiety was modelled over four positions. Both libration and disorder are unsurprising, as the ligands in both cases are bisected by mirror planes, whilst occupational disorder in abdc is ascribed to the lack of ligand mirror symmetry. Whilst the average structure is cubic and isostructural with UiO‐67, the local structure of the abdc ligand must break this symmetry (Figure 1 d). Interestingly, this disorder did not result in any observable diffuse scattering, which has been a point of great interest recently.^14^

Quantum mechanical simulations were performed on both UiO‐67 and UiO‐abdc using the crystallographic coordinates as starting models (Supporting Information, Section SI‐2). Motion of the six independent linker arms was followed by MD simulations, which revealed highly dynamic behavior. Atomic probability density functions (PDFs), analogous to thermal ellipsoid models in crystallographic refinements, were derived for the Zr_6_O_4_(OH)_4_ core, and one of the six linker units in each case (Figure 2).^15^ The resulting plots clearly demonstrate the extent of ligand movement observed across the horizontal mirror plane during the simulations (Supporting Information, Figure S2). A bowing angle, *θ*, was defined for each ligand (the angle that the benzene carboxylate makes with the Zr_4_ metal cluster square plane) and an average magnitude defined for both frameworks. Whilst the bpdc ligands in UiO‐67 remain approximately planar (〈|*θ*|〉=3(2)°), the geometric frustration in UiO‐abdc is accommodated by a more significant bowing of the ligands 〈|*θ*|〉=5(3)° (Figure 2). Good agreement between bond lengths in the time averaged MD and crystallographic models of both UiO‐67 and UiO‐abdc (Supporting Information, Table S1) is observed. The simulated/experimental overlay image of UiO‐67 (Figure 2 a) shows close alignment between the MD time‐averaged and crystallographic models. In a stark contrast, bowing of the abdc ligand on either side of the horizontal mirror plane is clearly observed in UiO‐abdc (Figure 2 b).

**Figure 2:**
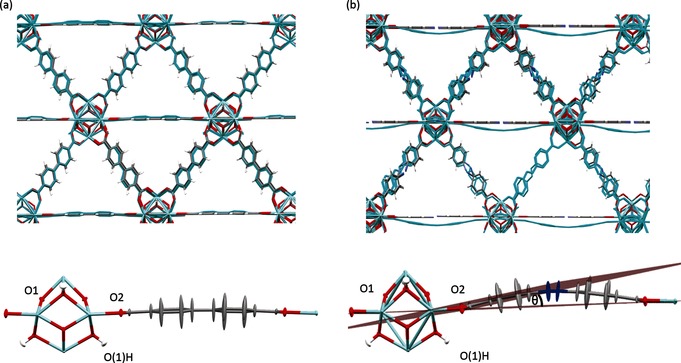
a) Top: Mean atomic positions model for UiO‐67 (in cyan) superimposed on the crystallographic structure. Bottom: Calculated atomic PDFs, drawn at the standard 50 % emphasizing thermal motion in the bpdc linker. b) Top: Mean atomic positions model for UiO‐abdc (in cyan) superimposed on the crystallographically disordered structure. Bottom: Calculated atomic PDFs, drawn at the standard 50 % emphasizing thermal motion in the abdc linker. Θ is defined by the intersection of a plane drawn through the equatorial Zr atoms and the carbon atoms of the first aromatic ring on the linker. C: grey, O: red, Zr: light blue, N: dark blue.

To investigate the effect of the higher flexibility and geometric frustration of abdc on the mechanical properties of the UiO framework, high‐pressure experiments were performed on both UiO‐67 and UiO‐abdc by loading suitable single‐crystals into modified Merrill–Bassett diamond anvil cells (DACs).^16^ In separate experiments, the MOF crystal was then surrounded by either methanol (MeOH) or fluorinert FC‐70 as the hydrostatic medium (Figure 3). Pore volume and content as a function of pressure were calculated using the SQUEEZE algorithm within PLATON (Supporting Information, Section SI‐4).^17^

**Figure 3:**
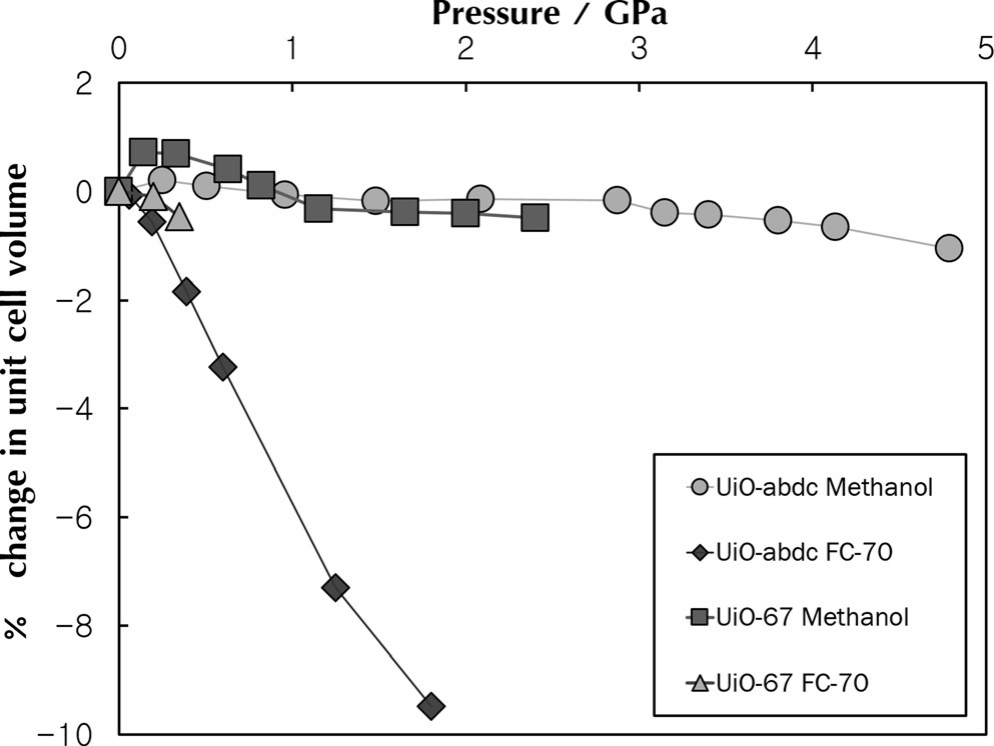
Graph of percentage change in volume vs. pressure (GPa) for UiO‐abdc in methanol (circles), FC‐70 (diamonds), and UiO‐67 in methanol (squares), FC‐70 (triangles).

On increasing pressure using MeOH as a hydrostatic liquid, both UiO‐67 and UiO‐abdc expand initially at 0.16 and 0.19 GPa, respectively (Figure 3). Such behavior has been observed in other compression studies of porous MOFs, where MeOH molecules penetrate into the solid and cause the framework to expand.^18^ On increasing pressure further, both frameworks begin to compress, before plateauing at 1.15 and 1.20 GPa in UiO‐67 and UiO‐abdc, respectively. UiO‐67 then remains almost incompressible to 2.4 GPa, while UiO‐abdc displays similar behavior to 4.8 GPa. The overall change in unit cell volume of the solvated UiO‐abdc of less than 1.2 % for such a large pressure regime is highly unusual. Experimental bulk moduli (*K*) were extracted from the experimental cell volume‐pressure relationships using EoS Fit (Supporting Information, Section SI‐4).^19^ Bulk moduli over a similar pressure range (0–2 GPa) of 174 GPa and 580 GPa for UiO‐67 and UiO‐abdc in MeOH were determined from the experimental data, though these numbers cannot be compared to existing literature data owing to the over‐solvated state of both frameworks. Nevertheless, the drastic change that inclusion of MeOH in the framework pores has on the compressibility is evident from these values, with the more porous MOF with the flexible abdc ligand being much more resilient to direct compression on inclusion of MeOH to much higher pressures than observed for the more rigid UiO‐67.

On initial pressurization using FC‐70 (a mixture of large perfluorinated hydrocarbons, usually considered a non‐penetrating hydrostatic medium) indirect evidence of guest inclusion can be observed due to an increase in compressibility observed in UiO‐67. On increasing pressure further, direct compression takes place in both frameworks. Unlike with MeOH, further increases in pressure are not accompanied by a plateau in cell volume. In fact, Bragg diffraction is lost from UiO‐67 at a relatively modest pressure of 0.3 GPa, yet UiO‐abdc undergoes a large change in unit cell volume of almost 10 % to 1.8 GPa, while remaining crystalline. Above 1.8 GPa, the quality of data resolution for UiO‐abdc was severely reduced, such that structural responses to increasing pressure could not be determined. The flexibility of the ligand in UiO‐abdc would, however, appear to impart a greater degree of resistance to increasing pressure whilst remaining crystalline.

To determine the compressibility of both frameworks without inclusion of the hydrostatic media, the mean atomic position structures obtained from the MD simulations were geometrically optimized by periodic DFT calculations, and then used as starting models for simulated hydrostatic compression in 0.2 GPa steps up to 1 GPa, thereby simulating direct compression experiments on guest‐free frameworks (Supporting Information, Figure S3). Compressions in cell volume of approximately 4.0 % and 6.0 % were observed at 1 GPa, respectively, for UiO‐67 and UiO‐abdc. While a 2^nd^ order Birch–Murnaghan equation of state (Supporting Information, Section SI‐4) allowed the determination of a bulk modulus of 16.8 GPa for UiO‐abdc; a step in the compression curve for UiO‐67 in the 0.2–0.4 GPa range prevents us from making a similar calculation. This hint of a structural transition could be linked to the loss of crystallinity observed experimentally in the same pressure range. These results are similar to previous computational work performed on evacuated MOF‐5, which reported 5 % compression at 1 GPa and a bulk modulus of 16.52 GPa.^18^ Consistent with the mechanical response of MIL‐type frameworks,^20^ the largest structural responses to the external pressure is observed for the ∡C‐O‐Zr‐Zr angle (*φ*) of the carboxylate functional group, which undergoes changes of up to 5° during compression of UiO‐abdc (Supporting Information, Figure S3).

For UiO‐abdc, the values of bulk moduli calculated from experimental data and calculations are in good agreement, with values of 14.8 GPa and 16.8 GPa, respectively. Because the early onset of pressure amorphization for UiO‐67 in FC‐70 precludes determination of *K*, and that value is similarly inaccessible from DFT under compression calculations, we turned to DFT calculations in the elastic regime (with infinitesimal strains). From the elastic tensors obtained (Supporting Information, Section SI‐7), the Voigt–Reuss bulk moduli calculated for UiO‐abdc is 15.2 GPa, in good agreement with the data above despite the very different methodology. The bulk modulus of UiO‐67 is slightly higher, at 17.4 GPa. Thus, the inclusion of the more flexible azobenzene‐based linker logically leads to a softer framework (higher compressibility), though the effect is quantitatively small. In comparison, the effect on the robustness or mechanical stability of the framework is much bigger: a six‐fold increase in its hydrostatic pressure at which it loses crystallinity.

To look at the response of the material under a different mechanical stimulation, we probed evacuated single crystals of UiO‐67 and UiO‐abdc by nanoindentation, to determine their Young's moduli, *E*, and hardness, *H*. Single‐crystal X‐ray diffraction was performed to establish Miller indices of the crystal facets (Supporting Information, Section SI‐5). Using the load‐displacement data (Supporting Information, Section SI‐6) gained during the indentation, *E* and *H* were calculated as a function of depth. The average values for each sample were calculated as *E=*20.02 GPa and *H*=1.27 GPa (UiO‐67), and *E*=13.24 GPa and H=0.65 GPa (UiO‐abdc). The trend seen in Young's moduli is in agreement with values derived from DFT calculations of elastic stiffness tensors, with UiO‐abdc again softer than UiO‐67 under uniaxial compression (24.1 and 21.5 GPa, respectively).

The elastic modulus of UiO‐67 is amongst the largest reported by nanoindentation for MOFs, and agrees with the low compressibility of the framework. The magnitude of this rigidity is, however, surprising given the empirical inverse relationship observed between *E* and framework solvent accessible volume (SAV), which for UiO‐67 is only moderately high (65.9 %; Supporting Information, Section SI‐8).^21^ Indeed, HKUST‐1 [Cu_3_(C_9_O_6_H_3_)_2_] has a similar SAV of 64.3 %, yet an elastic modulus of just 9.3 GPa.^22^ UiO‐67 is also markedly stiffer than the prototypical frameworks ZIF‐8 [Zn(C_3_H_3_N_2_)_2_] (*E=*3.15 GPa) and MOF‐5 [Zn_4_O(C_8_H_4_O_4_)_3_] (*E=*9.5 GPa), having SAVs of 50.3 % and 77.7 %, respect‐ively.^23^

The elastic modulus of UiO‐abdc is substantially lower than that of UiO‐67, which is in agreement with its higher SAV (71.8 %). It is interesting to note that this large decrease in rigidity is accompanied by a relatively small increase in SAV, whereas previous work on a different family of MOFs noted that changes in SAV of around 20 % would be required to elicit decreases in mechanical response of a similar order (ca. 40 %).23b This vastly more flexible nature is consistent with our observation of the frustrated, bowed nature of the abdc ligand in UiO‐abdc.

In conclusion, the different mechanical behavior of two UiO‐type frameworks has been fully characterized by computational and experimental methodologies. Bulk and elastic moduli for both UiO‐67 and UiO‐abdc demonstrate mechanical robustness.^24^ The near‐zero compressibility of UiO‐67 and especially UiO‐abdc when over‐solvated in MeOH is unique amongst the MOF world, and provides yet another example of the rich physical diversity of these systems, in addition to their much heralded chemical versatility. *E* in each case lies above those of other highly porous MOFs, and indeed approaches the mechanical response expected of dense hybrid frameworks.^25^ The large differences in elasticity with relatively small changes in SAV may allow fine‐tuning of mechanical response in these highly porous systems, though the effect of defects upon the properties of such materials remains an issue.14a, 26 The unexpected increase in resistance to pressure and the large decrease in the elastic modulus for UiO‐abdc compared to UiO‐67 are both ascribed to the presence of the azobenzene linker, which bows out of the horizontal plane. Similarly frustrated, bowed linkers cause significant disorder in other non‐UiO MOF structures,^27^ and as such may be a general phenomenon that subsequently impacts their mechanical behavior. These results are important for those looking to introduce flexibility and/or pressure‐coping mechanisms in other hybrid MOF systems.

## Supporting information

As a service to our authors and readers, this journal provides supporting information supplied by the authors. Such materials are peer reviewed and may be re‐organized for online delivery, but are not copy‐edited or typeset. Technical support issues arising from supporting information (other than missing files) should be addressed to the authors.

Supplementary

## References

[1a] H. Furukawa , K. E. Cordova , M. O'Keeffe , O. M. Yaghi , Science 2013, 341, 974–986; 10.1126/science.1230444 23990564

[1b] J. E. Mondloch , M. J. Katz , W. C. Isley III , P. Ghosh , P. Liao , W. Bury , G. W. Wagner , M. C. Hall , J. B. DeCoste , G. W. Peterson , R. Q. Snurr , C. J. Cramer , J. T. Hupp , O. K. Farha , Nat. Mater. 2015, 14, 512–516. 25774952 10.1038/nmat4238

[2] M. Bosch , M. Zhang , H.-C. Zhou , Adv. Chem. 2014, 2014, 1–8.

[3] T. D. Bennett , A. K. Cheetham , Acc. Chem. Res. 2014, 47, 1555–1562. 24707980 10.1021/ar5000314

[4a] D. Bazer-Bachi , L. Assié , V. Lecocq , B. Harbuzaru , V. Falk , Powder Technol. 2014, 255, 52–59;

[4b] G. W. Peterson , J. B. DeCoste , T. G. Glover , Y. G. Huang , H. Jasuja , K. S. Walton , Microporous Mesoporous Mater. 2013, 179, 48–53.

[5] Z. Li , H. C. Zeng , J. Am. Chem. Soc. 2014, 136, 5631–5639. 24635022 10.1021/ja409675j

[6a] I. E. Collings , M. G. Tucker , D. A. Keen , A. L. Goodwin , CrystEngComm 2014, 16, 3498–3506;

[6b] F. X. Coudert , Chem. Mater. 2015, 27, 1905–1916;

[6c] K. J. Gagnon , C. M. Beavers , A. Clearfield , J. Am. Chem. Soc. 2013, 135, 1252–1255; 23320490 10.1021/ja311613p

[6d] R. Matsuda , Nature 2014, 509, 434–435; 24848056 10.1038/509434a

[6e] S. A. Moggach , T. D. Bennett , A. K. Cheetham , Angew. Chem. Int. Ed. 2009, 48, 7087–7089; 10.1002/anie.200902643 19681088

[6f] L. Sarkisov , R. L. Martin , M. Haranczyk , B. Smit , J. Am. Chem. Soc. 2014, 136, 2228–2231. 24460112 10.1021/ja411673b

[7a] V. Bon , I. Senkovska , I. A. Baburin , S. Kaskel , Cryst. Growth Des. 2013, 13, 1231–1237;

[7b] S. J. Garibay , S. M. Cohen , Chem. Commun. 2010, 46, 7700–7702. 10.1039/c0cc02990d PMC3581306 20871917

[8] H. Wu , T. Yildirim , W. Zhou , J. Phys. Chem. Lett. 2013, 4, 925–930. 26291357 10.1021/jz4002345

[9] J. H. Cavka , S. Jakobsen , U. Olsbye , N. Guillou , C. Lamberti , S. Bordiga , K. P. Lillerud , J. Am. Chem. Soc. 2008, 130, 13850–13851. 18817383 10.1021/ja8057953

[10] A. Schaate , S. Dühnen , G. Platz , S. Lilienthal , A. M. Schneider , P. Behrens , Eur. J. Inorg. Chem. 2012, 790–796.

[11a] A. Schaate , P. Roy , A. Godt , J. Lippke , F. Waltz , M. Wiebcke , P. Behrens , Chem. Eur. J. 2011, 17, 6643–6651; 21547962 10.1002/chem.201003211

[11b] F. Vermoortele , B. Bueken , G. Le Bars , B. Van de Voorde , M. Vandichel , K. Houthoofd , A. Vimont , M. Daturi , M. Waroquier , V. Van Speybroeck , C. Kirschhock , D. E. De Vos , J. Am. Chem. Soc. 2013, 135, 11465–11468. 23875753 10.1021/ja405078u

[12] R. J. Marshall , S. L. Griffin , C. Wilson , R. S. Forgan , J. Am. Chem. Soc. 2015, 137, 9527–9530. 26175317 10.1021/jacs.5b05434

[13] B. Van de Voorde , I. Stassen , B. Bueken , F. Vermoortele , D. De Vos , R. Ameloot , J. C. Tan , T. D. Bennett , J. Mater. Chem. A 2015, 3, 1737–1742

[14a] M. J. Cliffe , W. Wan , X. D. Zou , P. A. Chater , A. K. Kleppe , M. G. Tucker , H. Wilhelm , N. P. Funnell , F. X. Coudert , A. L. Goodwin , Nat. Commun. 2014, 5, 4176; 24946837 10.1038/ncomms5176 PMC4730551

[14b] C. A. Trickett , K. J. Gagnon , S. Lee , F. Gándara , H.-B. Bürgi , O. M. Yaghi , Angew. Chem. Int. Ed. 2015, 54, 11162–11167; 10.1002/anie.201505461 26352027

[15] A. M. Reilly , S. Habershon , C. A. Morrison , D. W. H. Rankin , J. Chem. Phys. 2010, 132, 134511. 20387945 10.1063/1.3387952

[16] S. A. Moggach , D. R. Allan , S. Parsons , J. E. Warren , J. Appl. Crystallogr. 2008, 41, 249–251.

[17] A. L. Spek , J. Appl. Crystallogr. 2003, 36, 7–13.

[18] A. J. Graham , D. R. Allan , A. Muszkiewicz , C. A. Morrison , S. A. Moggach , Angew. Chem. Int. Ed. 2011, 50, 11138–11141; 10.1002/anie.201104285 22191090

[19] R. A. Angel , J. Gonzalez-Platas , M. Alvaro , Z. Kristallogr. 2014, 229, 405–419.

[20] C. Serre , S. Bourrelly , A. Vimont , N. A. Ramsahye , G. Maurin , P. L. Llewellyn , M. Daturi , Y. Filinchuk , O. Leynaud , P. Barnes , G. Ferey , Adv. Mater. 2007, 19, 2246–2251.

[21] M. W. Zhang , Y. P. Chen , M. Bosch , T. Gentle , K. C. Wang , D. W. Feng , Z. Y. U. Wang , H. C. Zhou , Angew. Chem. Int. Ed. 2014, 53, 815–818; 10.1002/anie.201307340 24218230

[22] S. Bundschuh , O. Kraft , H. K. Arslan , H. Gliemann , P. G. Weidler , C. Woll , Appl. Phys. Lett. 2012, 101, 101910.

[23a] J. Y. Jung , F. Karadas , S. Zulfiqar , E. Deniz , S. Aparicio , M. Atilhan , C. T. Yavuz , S. M. Han , Phys. Chem. Chem. Phys. 2013, 15, 14319–14327; 23877231 10.1039/c3cp51768c

[23b] J. C. Tan , A. K. Cheetham , Chem. Soc. Rev. 2011, 40, 1059–1080. 21221446 10.1039/c0cs00163e

[24] J. C. Tan , T. D. Bennett , A. K. Cheetham , Proc. Natl. Acad. Sci. USA 2010, 107, 9938–9943. 20479264 10.1073/pnas.1003205107 PMC2890448

[25] T. D. Bennett , J. C. Tan , S. A. Moggach , R. Galvelis , C. Mellot-Draznieks , B. A. Reisner , A. Thirumurugan , D. R. Allan , A. K. Cheetham , Chem. Eur. J. 2010, 16, 10684–10690. 20806296 10.1002/chem.201001415

[26] M. J. Cliffe , J. A. Hill , C. A. Murray , F. X. Coudert , A. L. Goodwin , Phys. Chem. Chem. Phys. 2015, 17, 11586–11592. 25866163 10.1039/c5cp01307k

[27a] V. Bon , I. Senkovska , M. S. Weiss , S. Kaskel , CrystEngComm 2013, 15, 9572–9577;

[27b] H. Furukawa , Y. B. Go , N. Ko , Y. K. Park , F. J. Uribe-Romo , J. Kim , M. O'Keeffe , O. M. Yaghi , Inorg. Chem. 2011, 50, 9147–9152. 21842896 10.1021/ic201376t

