# Supplementary material for: A Computational and Experimental Approach Linking Disorder, High‐Pressure Behavior, and Mechanical Properties in UiO Frameworks
Source: Angew Chem Int Ed Engl. 2016 Jan 21;55(7):2401–5. doi: 10.1002/anie.201509352 (PMC5021150; doi:10.1002/anie.201509352)
Supplement: Supplementary file 1 — Supplementary [file ANIE-55-2401-s001.pdf]

Angewandte Chemie
Eine Zeitschrift der Gesellschaft Deutscher Chemiker

Supporting Information

A Computational and Experimental Approach Linking Disorder, High-Pressure Behavior, and Mechanical Properties in UiO Frameworks

Claire L. Hobday, Ross J. Marshall, Colin F. Murphie, Jorge Sotelo, Tom Richards, David R. Allan, Tina Düren, François-Xavier Coudert, Ross S. Forgan, Carole A. Morrison,* Stephen A. Moggach,* and Thomas D. Bennett**

anie_201509352_sm_miscellaneous_information.pdf

Supporting Information

SI-1: General Materials and Methods

SI-2: Molecular Dynamics and DFT Calculations

SI-3: High Pressure DFT Geometry Optimisation Calculations

SI-4: High-Pressure X-ray Experiments

SI-5: Single crystal Face Indexing

SI-6: Nanoindentation Experiments

SI-7: Elastic Moduli

SI-8: Pore Volume Calculations

SI-1: Materials and Methods

Synthesis

UiO-67. $ZrCl_4$ (0.210 g, 0.90 mmol, 1 eq), biphenyl-4,4′-dicarboxylic acid (0.218 g, 0.90 mmol, 1 eq) and L-proline (0.500 g, 4.50 mmol, 5 eq) were added to a 50 ml screw top Pyrex jar. 20 ml of DMF was added then HCl (0.08 ml) was added. The reaction mixture was sonicated for several minutes until a homogeneous white suspension remained. The white suspension was transferred to an acid digestion vessel and sealed before being placed in the oven at 120°C for 24 hours. The reaction vessel was removed from the oven and allowed to cool to room temperature. The contents of the acid digestion vessel were removed by pipette and added to a 50 ml centrifuge tube. The reaction DMF was exchanged for fresh DMF several times. The crystals were retained in DMF before being analysed by single crystal X-ray diffraction (SCXRD).

UiO-abdc. $ZrCl_4$ (0.052 g, 0.225 mmol, 1 eq), 4,4-azobenzenedicarboxylic acid (0.061 g, 0.225 mmol, 1 eq) and L-proline (0.104 g, 0.900 mmol, 4 eq) were added to a 50 ml screw top Pyrex jar. 10 ml of DMF was added and the jar was placed in the sonicator for 5 minutes. HCl (0.02 ml) was added to the resulting suspension and the jar was placed in the sonicator for 5 minutes. The glass jar was placed in the oven at 100°C for 48 hours. The glass jar was removed from the oven and allowed to cool to room temperature. The reaction DMF was exchanged for fresh DMF several times. The crystals were retained in DMF before being analysed by SCXRD.

Face Indexing

Face-indexing measurements were performed at room temperature using an Oxford diffraction SuperNova X-ray source using Cu Kα_1 (λ =1.540598 Å) equipped with an Atlas detector. Data were collected in ω-scans in four settings of 2θ and φ with a step size of 1°. Exposure time for crystal UiO-67 was 60 s at low angle and 180s at high-angle. For crystal UiO-abdc exposure time was 1 s and 40 s at low and high angle respectively. Data processing, unit cell determination and face indexing were carried out using the program CrysAlisPro.

High Pressure X-ray Experiments

Compressibility studies were carried out on UiO-67 and UiO-abdc. The hydrostatic media chosen were methanol (MeOH) and fluorinert FC-70 for UiO-67 and UiO-abdc.

Firstly, an ambient pressure single crystal data collection was taken on both MOFs. Synchrotron high pressure single crystal diffraction experiments were carried out on two samples: UiO-67 with MeOH over a pressure range of 0.14 GPa to 2.40 GPa and UiO-abdc in fluorinert FC-70 from 0.06 GPa to 1.80 GPa. Laboratory high pressure single crystal measurements were carried out on UiO-67 in fluorinert FC-70 from 0.20 GPa to 0.35 GPa and UiO-abdc in MeOH over a pressure range of 0.19 GPa to 4.8 GPa.

Laboratory single crystal measurements. A single crystal was mounted onto a MiTiGen Microloops TM and a sphere of data were collected on a Bruker SMART APEX II diffractometer with graphite-monochromated Mo Kα radiation (λ= 0.71073 Å). These data were integrated using the program SAINT and the absorption correction was carried out using the program SADABS. [1]

Synchrotron radiation high pressure single crystal measurements. A single crystal was loaded into a Merrill-Bassett diamond anvil cell (DAC) with a half-opening angle of 40 degrees, composed of Boehlar Almax diamonds with 600μm culet diamond anvils, a tungsten gasket and tungsten carbide backing plates.[2] The appropriate hydrostatic pressure medium was added. A small ruby chip was also loaded into the cell to act as a pressure calibrant, using the pressure dependent fluorescence of the ruby to measure the pressure.[3] Diffraction data were collected on station I19 at the DIAMOND Light Source, Rutherford Appleton Laboratory, on a Huber 4-circle goniometer with a Rigaku Saturn 724 CCD detector using synchrotron radiation (wavelength = 0.5159 Å) from 0.14 GPa to 3.8 GPa. Data collection was carried out using an exposure time and a step size of 1 second and 0.5 degrees respectively. The data were integrated with the program SAINT using dynamic masks (these mask the regions of the detector which are shaded due to the pressure cell).[1a, 4] Omission of shaded reflections, absorption correction and merging of data were carried out in a three-step process, firstly with the program SHADE, then SADABS and finally XPREP.[1b, 5]

Laboratory high pressure single crystal measurements. A suitable single crystal was placed in a Merrill-Bassett DAC composed of a tungsten gasket, 600µm diamond culets and tungsten carbide backing discs with a half opening angle of 40 degrees.[2] The appropriate hydrostatic pressure medium was added and small ruby chip was also loaded into the cell to act as a pressure calibrant, as previously described. High pressure diffraction data were collected on a Bruker APEX II diffractometer with graphite-monochromated Mo Kα radiation (0.71073 Å). Data were collected from 0.1 GPa to 0.9 GPa in omega scans in eight settings of 2θ and φ with an exposure time of 100 s and a step size of 0.5°. The data were integrated using dynamic masks and absorption corrections for the DAC and sample were carried out with the programs SHADE and SADABS, respectively. [1, 4-5]

Structure refinements. Structure refinements were carried out in CRYSTALS.[6] The structure was solved by SUPERFLIP.[7] All structures were refined anisotropically against *F* with an I/σ cut-off of -3, to reject weak reflections. All 1,2 and 1,3 distances for the organic linker (biphenyl dicarboxylate or azobenzenedicarboxylate for UiO-67 and UiO-abdc respectively) were restrained, whilst all torsion angles and metal – ligand bond distances were allowed to freely refine. Vibrational and thermal similarity restraints were also applied to the organic linker. Hydrogen atoms on the linker were placed geometrically and constrained to ride on their host atoms. The pore volume and electron count per unit cell were calculated using the SQUEEZE algorithm within PLATON. [8] The data collected at high-pressure for UiO-67 in MeOH and FC-70 was not of sufficient quality to refine a satisfactory structure above 2.4 GPa and 0.35 GPa respectively. For UiO-abdc, structures could only be obtained up to 0.39 GPa in FC-70. Only unit cell dimensions could be extracted for the laboratory data collected on UiO-abdc in MeOH.

Nanoindentation. Nanoindentation experiments were performed using an MTS Nanoindenter XP, located in an isolation cabinet to shield against thermal fluctuations and acoustic interference. Samples were first mounted using an epoxy resin, which was then ground away using a silicon carbide disc, before being polished using increasingly fine diamond suspensions. Indentations were conducted under the dynamic displacement-controlled "continuous stiffness measurement" mode. *E* (Young's Modulus) and *H* (Hardness*)* were subsequently determined as a function of the surface penetration depth. A 2-nm sinusoidal displacement at 45 Hz was superimposed onto the system's primary loading signal, and the loading and unloading strain rates were set at $5{\times}10^{-2}\ s^{-1}$. All tests were performed to a maximum indentation depth of 500 nm (unless otherwise stated) using a

Berkovich (i.e., three-sided pyramidal) diamond tip of radius ~ 100nm. The raw data (load-displacement curves) obtained were analyzed using the Oliver and Pharr method. [9]

SI-2: Molecular Dynamics Calculations

All ab initio (Born-Oppenheimer) MD calculations were performed using the Quickstep module of the CP2K (version 2.6) simulation package.[10] The BLYP[11] exchange-correlation functional with semi-empirical dispersion corrections to the energies and gradients from the DFT-D3[12] method (cut-off radius 10 Å) where used throughout. Energies and forces were calculated utilizing the Gaussian plane-wave scheme, which is a dual basis set method wherein a linear combination of Gaussian-type orbitals is used to describe the Kohn-Sham molecular orbitals while the electron density is described by an auxiliary plane-wave basis set (expressed at an energy cut-off of 350 Ry, accompanied by the relative cutoff of 50 Ry for the Gaussian basis set collocation). The double-zeta quality MOLOPT basis set [13] was used for all elements, in conjunction with the relativistic, norm-conserving Goedecker-Teter-Hutter pseudopotentials, optimized for use against the BLYP functional. During each SCF cycle, the electronic structure was explicitly minimized to a tolerance of 10^{-7} Hartree. The equations of motion were integrated using a time step of 0.55 fs.

The crystallographic models of both UiO-67 and UiO-abdc were recast to their primitive cell settings, thereby reducing the volume of the crystallographic unit cells to a quarter of their conventional setting, and presenting a considerable cost-saving for the modelling work. However, this still resulted in relatively large unit cell models (a = b = c= *ca*. 20 Å, α = β= γ = 60°), which by definition results in compact 1st Brillouin zones. Thus the constraint that the QUICKSTEP module employs Γ-point sampling only of the Brillouin zone was not a concern in this work. For the crystallographically disordered UiO-abdc, equilibration of the model was initiated under the isobaric-isothermal ensemble regime (NPT; constant number of particles, pressure and temperature) for 4 ps. The temperature was set to 300 K and controlled by a chain of Nosé-Hoover thermostats[14] coupled to every degree of freedom (the so-called massive thermostat) with a frequency of 4000 cm^{-1}, which is high enough to properly sample the fast vibrational motion of the O-H bond in the ZrO cluster of the UiO-67-based MOFs. The barostat was set up with a coupling time constant of 300 fs and an external pressure of 1 bar. In addition, a reference unit cell of constant volume was defined alongside the model of UiO-abdc to fix the number of grid points used to compute the Coulomb and exchange-correlation energies. This was used to mitigate any effects of varying grid points due to potential volume fluctuations of the simulation box (due to large breathing motions of UiO-abdc). It has been shown previously that the use of such a reference cell avoids any discontinuities in the potential energy profile when the volume is permitted to vary.[15] However, minimal (2 %) unit cell expansion was observed by this process. The unit cell

parameters were then fixed at the equilibrated values, and the ensemble switched to NVT for production run dynamics (4 ps). For UiO-67, with rigid linker geometry even less unit cell expansion was anticipated; for this reason system equilibration (1 ps) and production run dynamics (4 ps) were all performed in the NVT ensemble (with same settings as listed above for UiO-abdc). The resulting trajectories were then analysed numerically to determine the time-averaged mean atomic positions(which are simply the coordinates for each atom averaged over all frames from the production run data set), and to calculate the atomic probability density functions (via numerical calculation of the variances and co-variances of each atom, using methods described previously).[16] The later are analogous to the thermal ellipsoid model used in crystallographic refinements, and are displayed in in the main text at the standard 50% probability level. The output data were then processed graphically using Mercury CSD 3.3.1.[17] The time-average atomic positions were suitable models to use as starting points for geometry optimization calculations (reported in the following section). Selected geometrical parameters from the time averaged structures are given in Table S1, alongside the corresponding values from the crystallographic study.

Table S1: Selected parameters from the experimentally derived crystal structures of UiO-abdc and UiO-67, at 0 and 1 GPa, compared with the time-averaged structures (at 1GPa) derived from ab initio molecular dynamics.

	UiO-abdc			UiO-67		
**Parameters r/Å, ∠/°.**	Exp (0 GPa)	Exp (1 GPa)	Time-averaged Calc[c]	Exp (0 GPa)	Exp (1 GPa)	Time-averaged calc
**Av. Zr-Zr**	3.473(3)	3.504(3)	3.572(6)	3.5056(2)	3.50(6)	3.583(3)
**Av. Zr-O1H/ Zr-O1 $_{(cluster)}$**[a]	2.119	2.256(2) 2.058(1)	2.296(5) 2.092(3)	2.1369	2.2307 2.073(12)	2.301(2 2.098(1)
**Av. Zr-O2 $_{(ligand)}$**[a]	2.208	2.192(43)	2.248(4)	2.2246	2.192(26)	2.262(3)
**Av. O-C**	1.268	1.272(3)	1.276(7)	1.2716	1.274(2)	1.280(1)
**Av. C-C**	1.505	1.4676(8)	1.473(10)	1.4623	1.4700(5)	1.46(4) 1.489(3)
**Av. C_{arom}-C_{arom}**	1.388(7)	1.393(11)	1.371(15)	1.3639(17)	1.399(21)	1.36(3)
**Av. C-N/C-C**	1.429 /-	1.3938(9)/ -	1.413(9) /-	- /1.5116	- /1.4665(7)	-/1.487(21)
**Av. N-N**		1.2926(1)	1.219(16)	-	-	-
**θ**	0	6.99(2.6)	5(3)	0	0	3(2)

[a] See Fig. 2 in main text for atomic labels. [c]Parameters obtained from averaging atomic coordinates (*P*1 model) over production run Molecular Dynamics trajectory (NPT: 1 GPa, 300 K).

The cage Zr-Zr and Zr-O2 simulated bond distances are also in excellent agreement (to within 0.05 Å) with work reported by Valenzano *et al* for the structurally related UiO-66 using EPR spectroscopy and X-ray powder diffraction.[18] The time averaged MD structure returned noticeably different values for the Zr-O1 cluster distances, depending whether or not the μ^3-O(1) atoms were capped with hydrogen. The simulation suggests that capping the μ^3-O(1) atoms with hydrogen introduces a significant distortion to the ZrO cluster, with the oxygen atoms being pulled out from the cage (Figure S1). While this finding could not be substantiated by the best-fit space group assigned from the crystallographic data in this work (*Fm*-3*m,* due to the inability to locate the hydroxyl hydrogen atoms, and thus rendering all Zr-O(1) distances equivalent to one another), it is substantiated by simulation

work by Valenzano *et al*, and their equivalent Zr-O(1)H and Zr-O(1) distances agree with ours to within 0.01 Å.

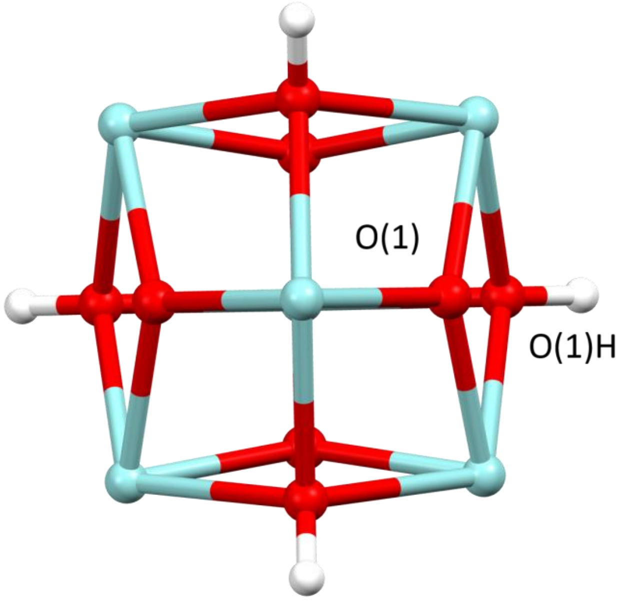

Figure S1: Time-averaged mean atomic positions for the $Zr_6O_4(OH)_4$ cluster derived from the MD trajectory highlighting two O(1) environments. Zr-O(1) and Zr-O(1)H distances are ~ 2.10 Å and 2.31 Å, respectively (C- grey, O – red, Zr –light blue, N – dark blue, H - white).

In order to define the degree of ligand flexibility observed in the molecular dynamics simulations a 'bow' angle, θ (defined as the deviation from 90°, as shown in Figure S2 of the main text) was derived for each frame from the trajectories simulated for both UiO-abdc and UiO-67. Plots showing the variation in this parameter for each of the six ligands for both framework systems is shown in Figure 2 below. From this it is readily apparent that UiO-abdc supports significantly more ligand flexibility, with half of the ligands flexing above and below the horizontal mirror plane (marked by the θ = 0° horizontal lines). The absence of such behavior for the other three ligands is in all probably a sampling issue: if the dynamic trajectory was run for a longer time this behavior would more than likely be observed for all ligands in UiO-abdc . The mean average value observed for UiO-abdc in this MD trajectory is 5(3)°. For UiO-67 ligand flexing was also observed but to a lesser degree, returning a mean average value of θ = 3(2)°.

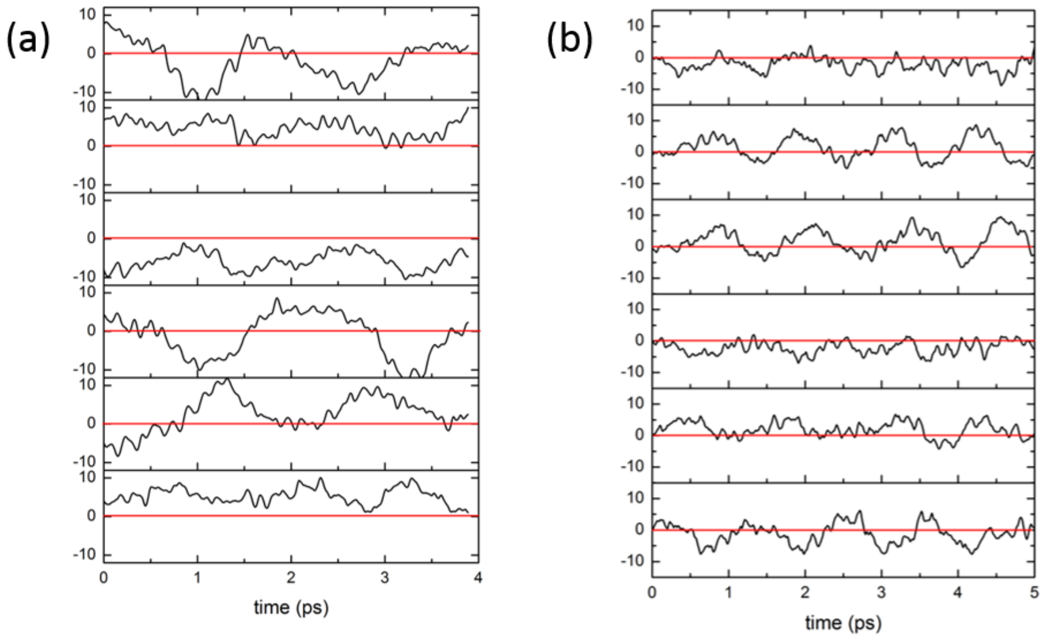

Figure S2: Plots showing the variation in θ against time for the six ligands in (a) UiO-abdc, (b) UiO-67.

SI-3: High Pressure DFT Geometry Optimisation Calculations

All calculations were performed using the CASTEP (version 5.11) simulation package.[19] The Hamiltonian operator was approximated using the Perdew-Burke-Ernzerhof (PBE) exchange-correlation functional, with the molecular wavefunction description provided by 'on-the-fly' pseudopotentials and a plane wave basis set operating at 650 eV, which gave convergence to within 4 meV per atom. In addition, a Tkatchenko-Scheffler dispersion correction was applied.[20] The electronic structure was sampled at the gamma position only in the Brillouin zone due to the large size of the primitive unit cell (resulting in a k-point sampling grid of no greater than 0.06 $Å^{-1}$). The geometry optimisation calculations, which took the time-averaged mean atomic position models derived from the MD production trajectories as input, were run without any symmetry constraints, thus allowing for an independent variation of both the atomic positions and the primitive cell parameters. The potential energy surface was searched for energy minima by means of the Broyden-Fletcher-Goldfarb-Shanno (BFGS) algorithm.[21] Structures were considered to be optimised when the energy per atom, maximum force, maximum stress, and maximum atomic displacement converged to the values of 0.02 meV/atom, 0.05 eV/Å, 0.1 GPa, and 0.002 Å, respectively. Once the

first, ambient pressure, model was optimised, an external hydrostatic pressure of 0.2 GPa was applied and the system re-optimised; this process was repeated at 0.2 GPa steps until an external pressure of 1 GPa was reached (Fig 3). After 1 GPa, the optimization of UiO-abdc could not be achieved as the structure had reached the limit of sensible bonding.

Results obtained from the direct compression simulations are shown in Fig S3.

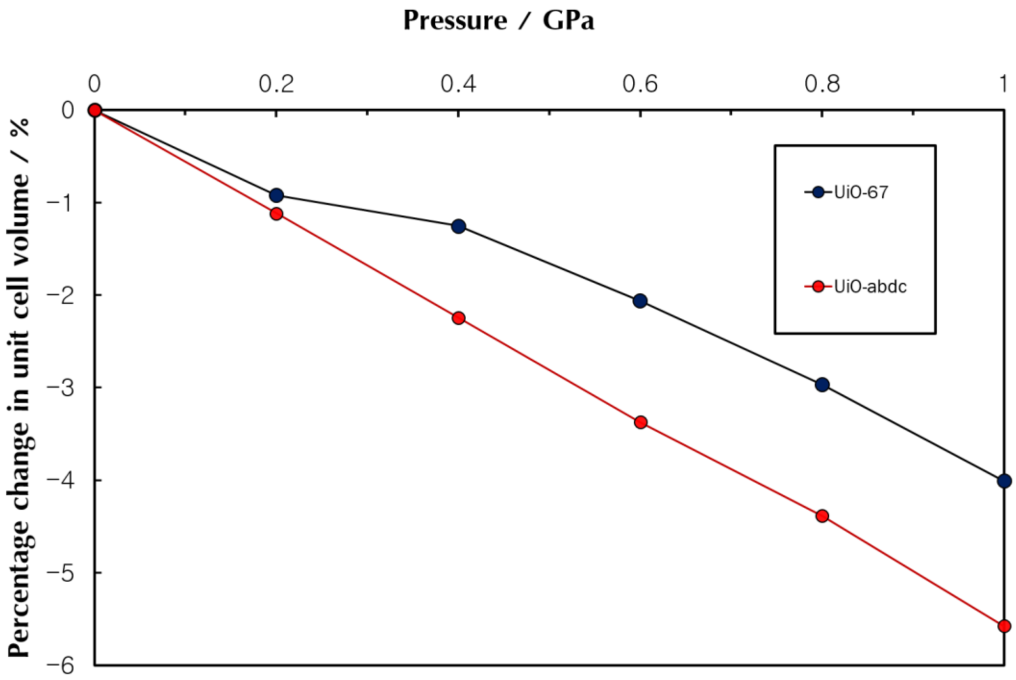

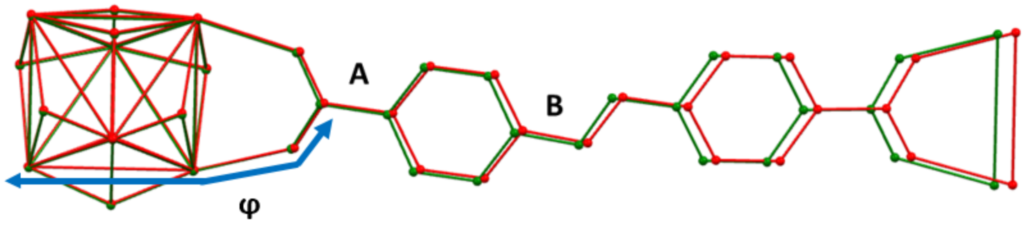

Figure S3: (Top) DFT simulated direct compressibility of UiO-67 and UiO-abdc. (Bottom) overlay of part of the DFT optimized ambient (red) and 1 GPa (green) structures of UiO-abdc, highlighting the, ∠C-O-Zr-Zr angle (ϕ), in addition points A and B which undergo the most compression in the system to allow for such linear compressibility.

Comparing the computational compression with the high pressure FC-70 compression of UiO-abdc (see Fig. S4), we see that the computational models the experiment well.

However, at low pressures we see that the DFT study predicts that UiO-abdc to compress more than in FC-70, but at higher pressures the rate of compression is equal. This initial resistance to compression from the FC-70 experiments is thought to be inclusion of FC-70 at low pressures which is then expelled at higher pressures.

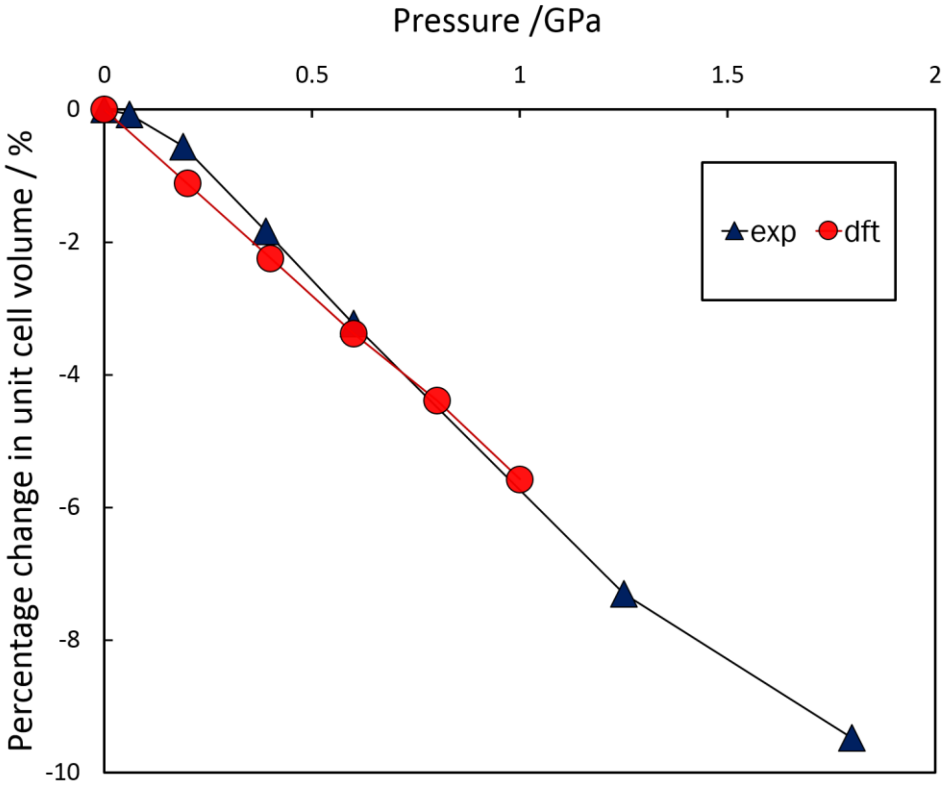

<u>Figure S4:</u> Graph of pressure vs change in unit cell volume for UiO-abdc. Red circles: DFT compression study and blue triangles are the experimental values in FC-70.

SI-4: High Pressure X-ray Experiments

Table S2: High pressure crystallographic data of UiO-67 in methanol (including squeeze output where applicable.)

Pressure [GPa]	Cell Volume [Å^3]	Total Pore Volume [Å^3]	Electron Count [e^-]
0.14	19511.463	13544	7567
0.33	19505.404	13522	4781
0.82	19391.545	13414	7636
1.15	19309.584	13336	6986
1.65	19296.057	13322	7423
2.00	19293.104	13306	8330
2.40	19277.396	13289	9368

Table S3: High pressure crystallographic data of UiO-67 in Fluorinert FC-70 (including squeeze output where applicable.) Note; these values are only tentative, as the data were of poor quality and resolution (less than 1.5 Å).

Pressure [GPa]	Cell Volume [Å^3]	Total Pore Volume [Å^3]	Electron Count [e^-]
0.2	19352.877	13444.1	3440
0.35	19376.658	13375.6	3738

Table S4: High pressure crystallographic pressure / volume data of UiO-abdc in methanol

Pressure [GPa]	Cell Volume [Å^3]
0.19	26302.416
0.40	25797.953
1.20	25787.715
1.75	25826.748
2.25	25824.207
2.45	25850.371
3.15	25773.561
3.40	25765.988
3.80	25728.357
4.80	25658.201

Table S5: High pressure crystallographic data of UiO-abdc in Fluorinert FC-70 (including squeeze output where data quality allows.)

Pressure [GPa]	Cell Volume [Å^3]	Total Pore Volume [Å^3]	Electron Count [e^-]
0.06	25853.721	19534	10203
0.19	25727.857	19417	10008
0.39	25395.09	19159	8818
0.6	25036.865	n/a	n/a
1.25	23984.266	n/a	n/a
1.8	23421.395	n/a	n/a

Using the experimental pressure/volume data, bulk modulus (K) values could be assigned to the material at specific pressure ranges. Whilst this is not an ideal approach, it does give a comparable approach to quantifying how incompressible UiO-abdc becomes when super-filled with methanol, compared to UiO-67. It is clear from this method that the UiO-abdc is extremely incompressible compared to UiO-67 over a larger pressure range. To directly compare UiO-67 and UiO-abdc in the solvated methanol, a fit was taken over a similar pressure range for both (see Fig. S4 and Fig. S6). A Birch Murghanhan equation of state fit is not possible as in a single crystal high-pressure study we are limited in the number of pressure/volume data points. To overcome this, a computational pressure study was carried out as well to effectively quantify the differences in compressibility as more gradual changes in pressure are allowed with the apparatus.

<u>Table S6</u>: K and related parameters for UiO-abdc compression in methanol

Pmin (GPa)	Pmax (GPa)	V_0 (Å^3)	K (GPa)	K'	K''	W chi^2
0	0.25	25876	-129.008	4	0	0
0.25	0.5	25947.6348	266.156	4	-0.0141	0.52
0.5	0.95	25943.9043	286.4097	4	-0.0136	0.03
0.95	1.48	25910.2148	465.9305	4	-0.0083	0.19
1.48	2.08	25800.6406	-1426.56	4	0.0027	0.05
2.08	2.875	25861.5039	2311.691	4	-0.0017	0
2.875	3.15	26410.0195	123.358	4	-0.0315	1.07
3.15	3.4	25900.0312	634.0259	4	-0.0061	0.04
3.4	3.8	26000.6035	363.7519	4	-0.0107	0.02
3.8	4.13	26120.8086	248.7943	4	-0.0156	0.07
4.13	4.79	26409.6426	144.5562	4	-0.0269	0
0	2.1	25923.1836	580.2522[a]	4	-0.0067	0.36
0	5.2	25943.0254	412.88[b]	4	-0.0094	0.42

[a]Fit of UiO-abdc over similar region as UiO-67 for comparison of compressibility in MeOH over similar range, shown in Fig. S5a. [b]Fit of UiO-abdc in MeOH for all pressure region, shown in Fig. S5b.

a)

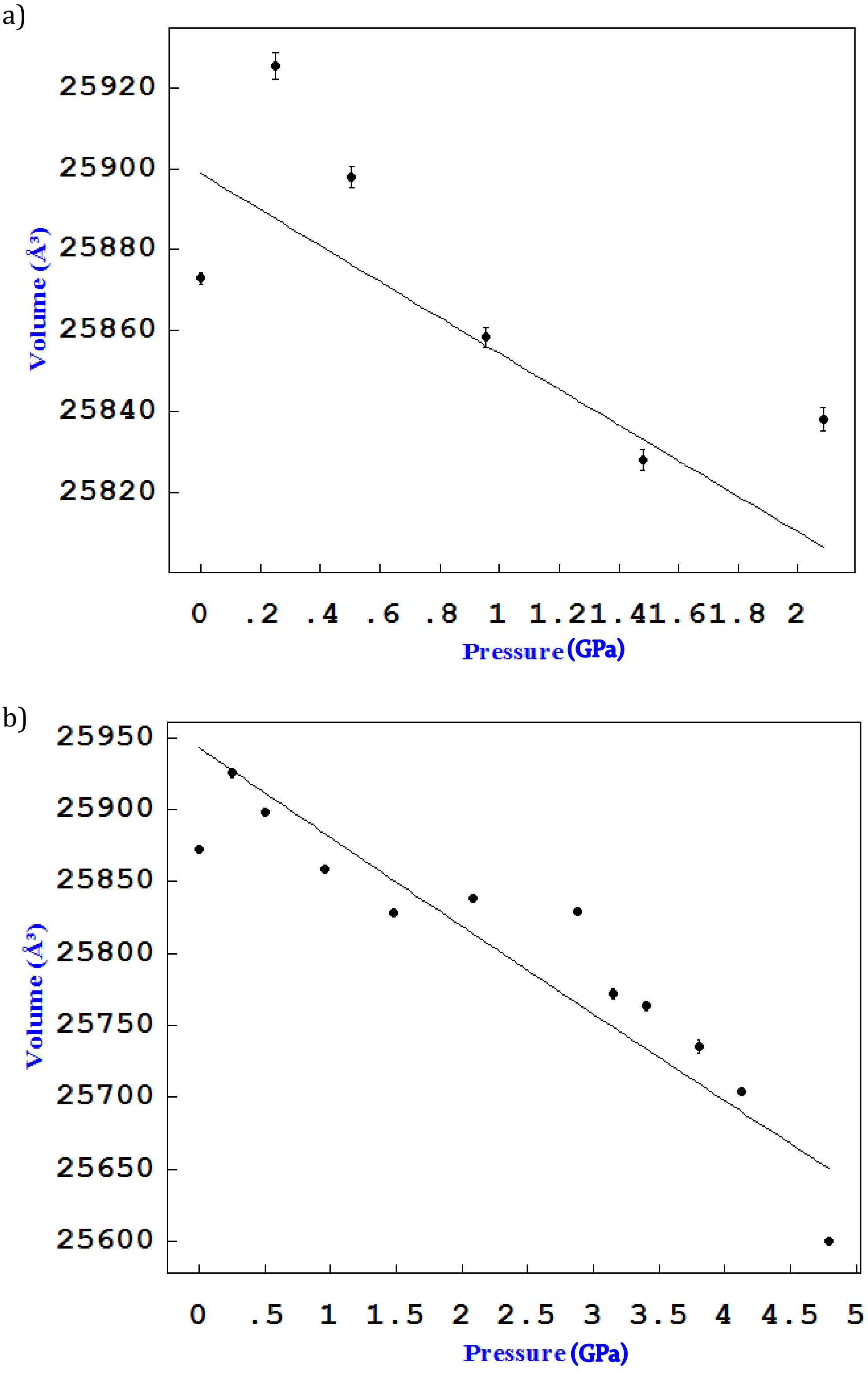

b)

Figure S5: a) Fit of UiO-abdc in MeOH over pressure range 0 GPa to 2.1 GPa to yield K of 580 GPa. b) Fit of UiO-abdc in MeOH over pressure range 0 GPa to 4.8 GPa, to yield K of 412 GPa.

Table S7: K and related parameters for UiO-67 compression in methanol

Pmin (GPa)	Pmax (GPa)	V_0 (Å^3)	K (GPa)	K'	K''	W chi^2
0	0.14	19398.33	-24.3488	4	0.1597	0.4
0.14	0.33	19516.07	601.5632	4	-0.0065	0
0.33	0.63	19570.44	99.9858	4	-0.0389	0.16
0.63	0.82	19640.87	62.7791	4	-0.0619	0.17
0.82	1.15	19600.24	74.7791	4	-0.052	0.03
1.15	1.65	19343.58	661.6235	4	-0.0059	0.04
1.65	2	19317.08	1566.016	4	-0.0025	0.08
2	2.4	19380.44	442.7786	4	-0.0088	0.09
0.33	1.15	19587	79.4156[c]	4	0.049	4.37
1.15	2.4	19340	749.046[d]	4	-0.0052	0.75
0	2.4	19481.53	174.67[e]	4	-0.0223	0.21

[c]Fit of UiO-67 from 0.33-1.15 GPa shown in Figure S7a, [d]fit of UiO-67 from 1.15-2.4 GPa shown in Figure S7b [e]fit of UiO-67 from 0 -2.4 GPa shown in Figure S7c

a)

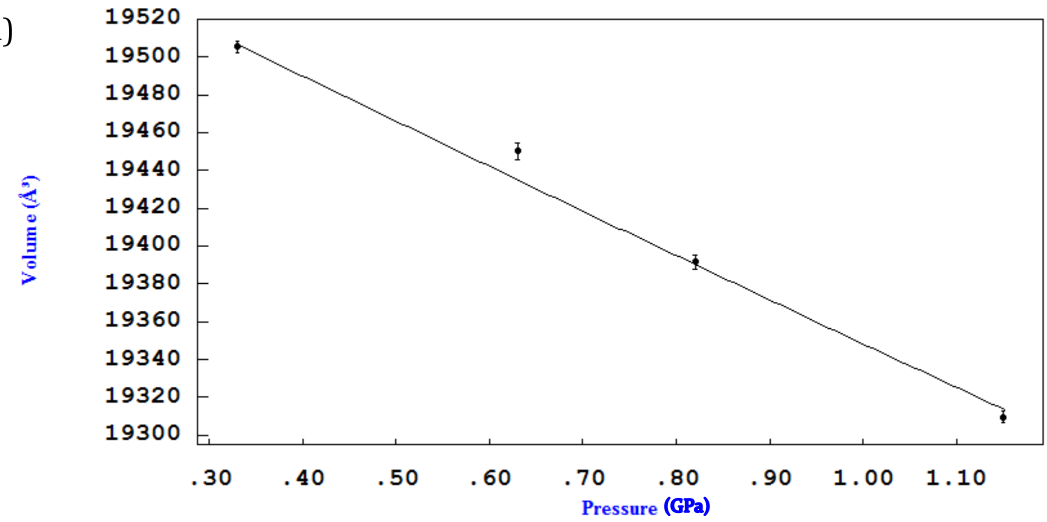

b)

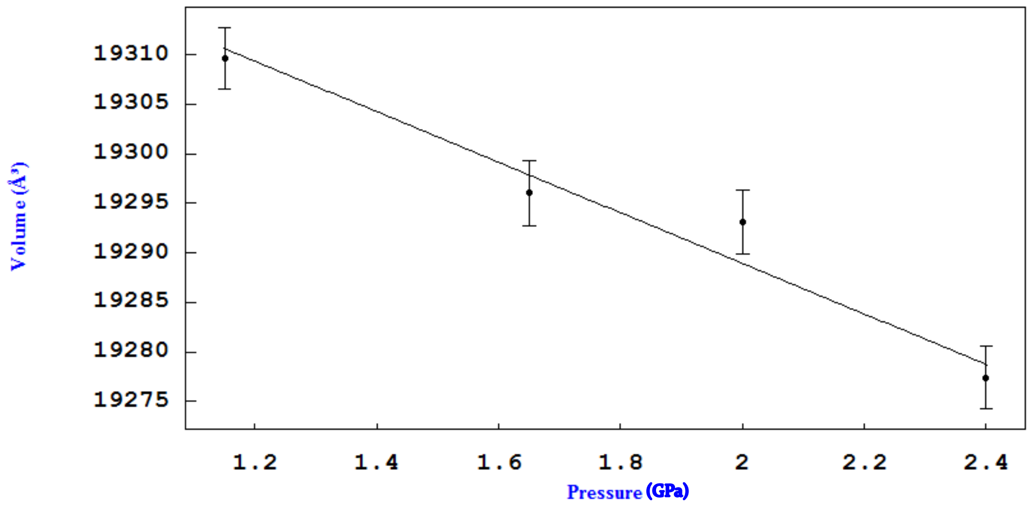

c)

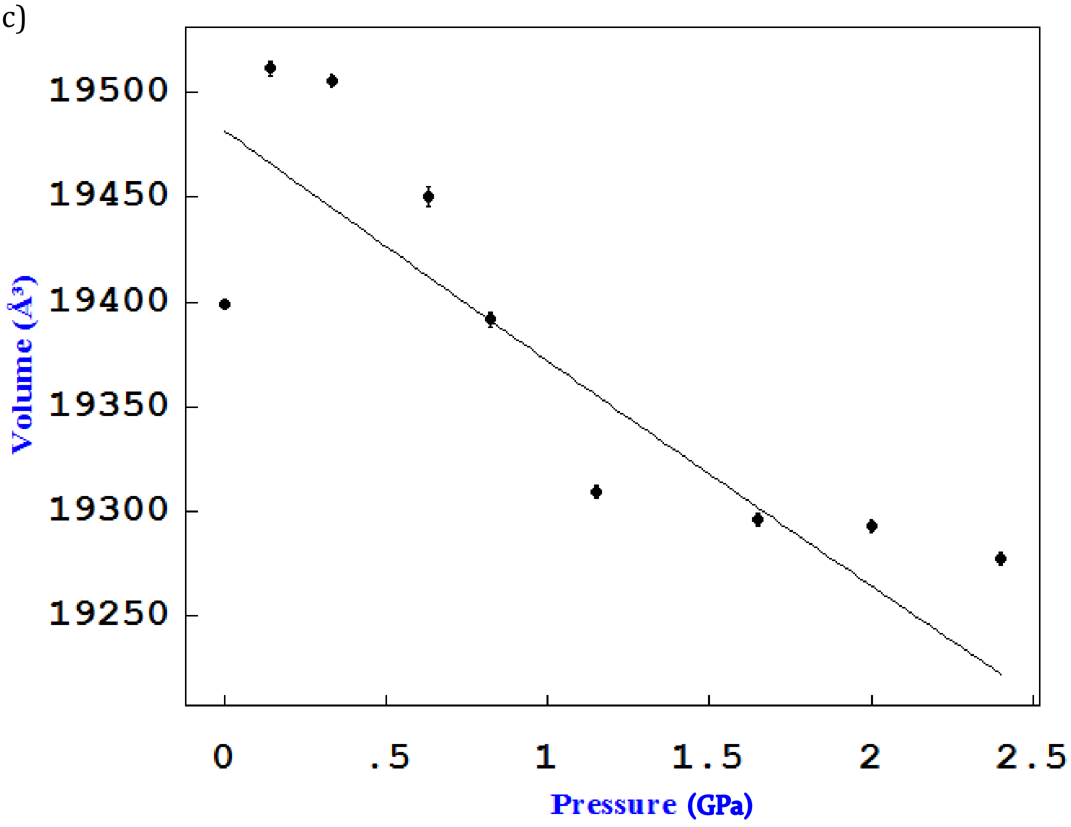

<u>Figure S6</u>: a) Fit of UiO-67 over pressure range 0.33 GPa to 1.15 GPa to yield K of 79 GPa. b) Fit of UiO-67 over pressure range 1.15 GPa to 2.4 GPa, to yield K of 749 GPa. c) Fit of UiO-67 over pressure range 0 GPa to 2.4 GPa, to yield K of 174.67 GPa.

Table S8: K_0 and related parameters for UiO-abdc compression in FC-70

Pmin /GPa	Pmax /GPa	V_0 /Å^3	K /GPa	K'	K''	W chi^2
0	0.25	25872.8574	14.8334	4	0.2622	0.01

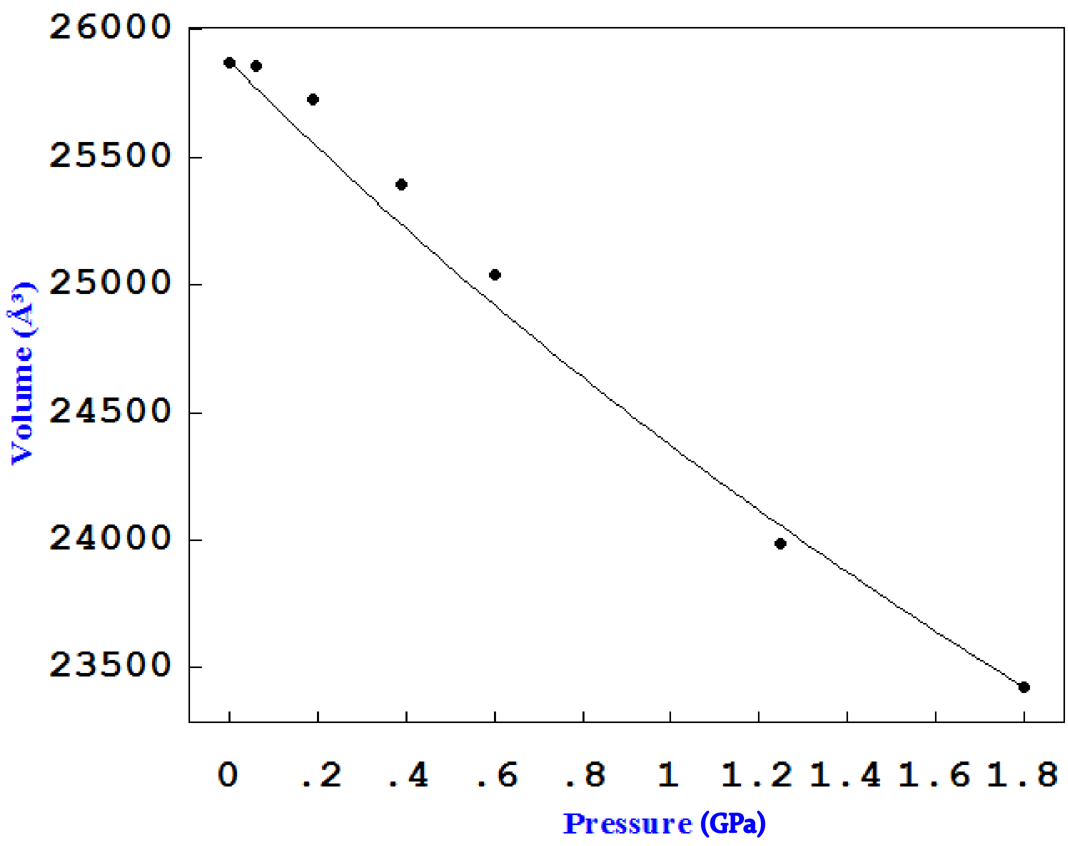

Figure S7: a) Fit of UiO-abdc in FC-70 over pressure range 0 GPa to 1.8 GPa to yield K of 14.83 GPa.

Table S9: Bulk Modulus Fit of UiO-67 and UiO-abdc from DFT compression study

	Pmin (GPa)	Pmax (GPa)	V_0 (Å^3)	K (GPa)	K'	K''
UiO-67	0	1.00	4956.0811	25.0694	4	-0.15512
UiO-abdc	0	1.00	6524.6201	16.8166	4	-0.2313

a)

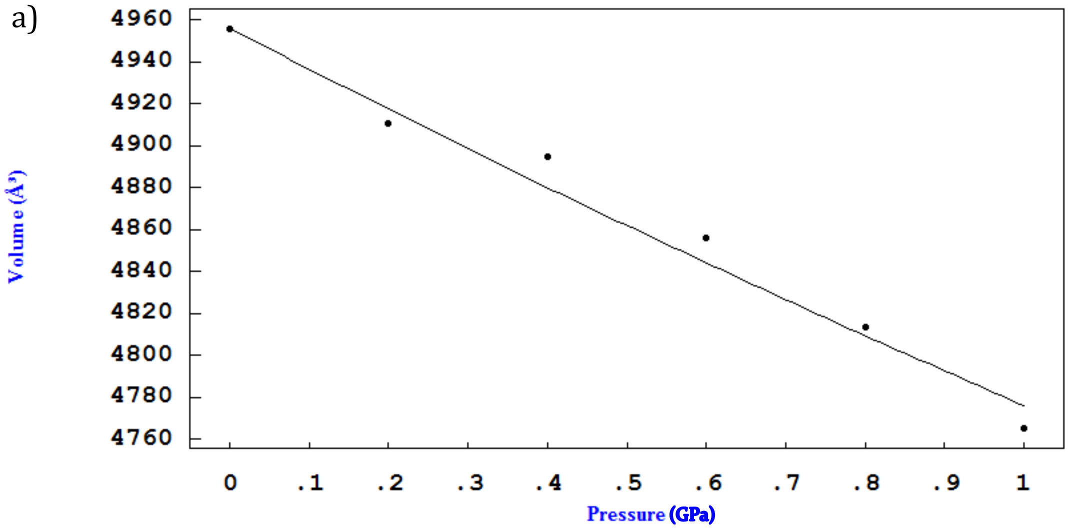

b)

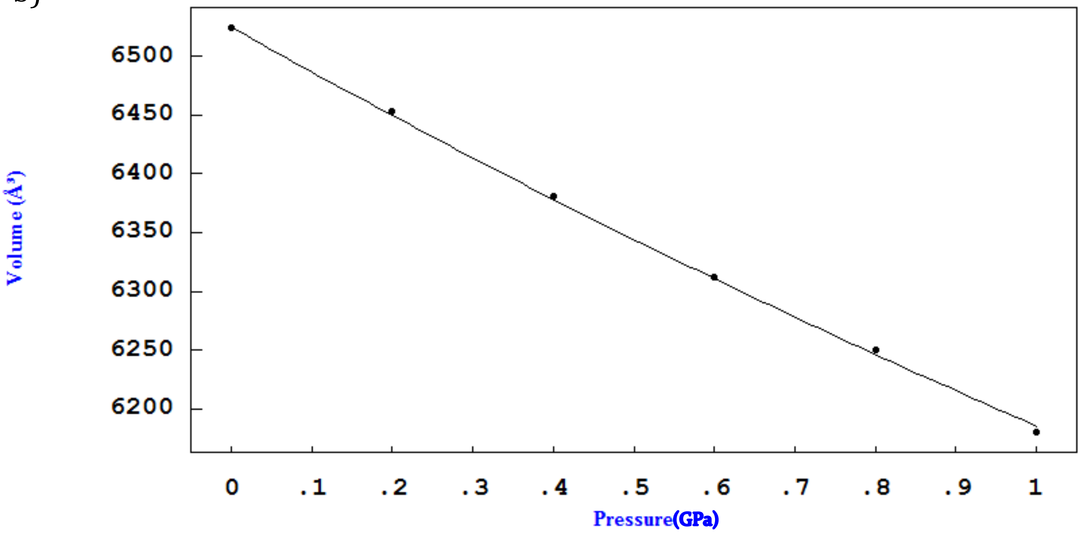

Figure S8: a) Fit of DFT optimized UiO-67 over pressure range 0 GPa to 1 GPa, to yield K of 25 GPa b) Fit of DFT optimized UiO-abdc over pressure range 0 GPa to 1 GPa, to yield K of 16.8 GPa

SI-5: Face Indexing

UiO-67

**Crystal system**	Cubic	
**Space group**	*Fm*-3*m*	
**Unit cell dimensions**	a = 26.8042(2) Å	α = 90°.
	b = 26.8042(2) Å	β = 90°.
	c = 26.8042(2) Å	γ = 90°.
**Volume**	19257.9(3) $Å^3$	
**Crystal size**	0.049 x 0.061 x 0.071 mm	

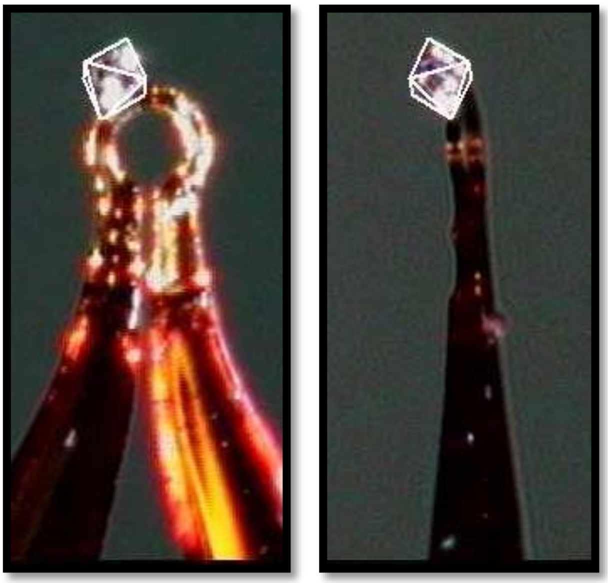

Figure S9: Side on and front views of the octahedral shaped crystal

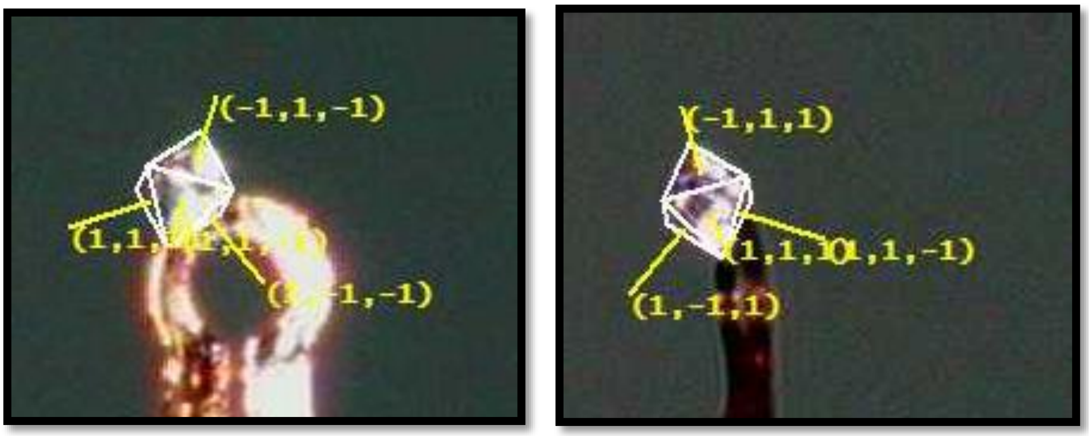

Figure S10: Face-normals of principal faces of the crystal, note the triangular shaped faces (1 1 1), (-1 1 1), (1 -1 1), (1 1 -1), (1 -1 -1), (-1 1 -1), (-1 -1 1) and (-1 -1 -1)

UiO-abdc

**Crystal system**	Cubic	
**Space group**	*Fm*-3*m*	
**Unit cell dimensions**	a = 29.4065(2) Å	α = 90°.
	b = 29.4065(2) Å	β = 90°.
	c = 29.4065(2) Å	γ = 90°.
**Volume**	25429.2(3) $Å^3$	
**Crystal size**	0.047 x 0.062 x 0.073 mm	

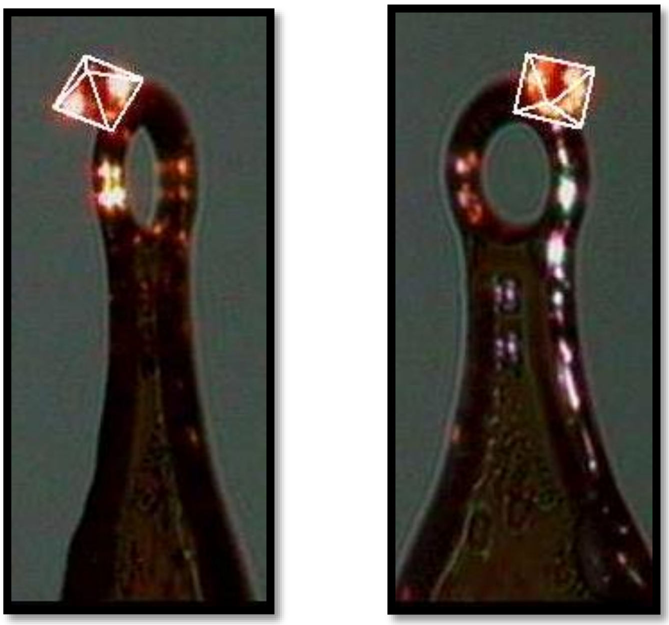

Figure S11: Side on and front views of the octahedral shaped crystal

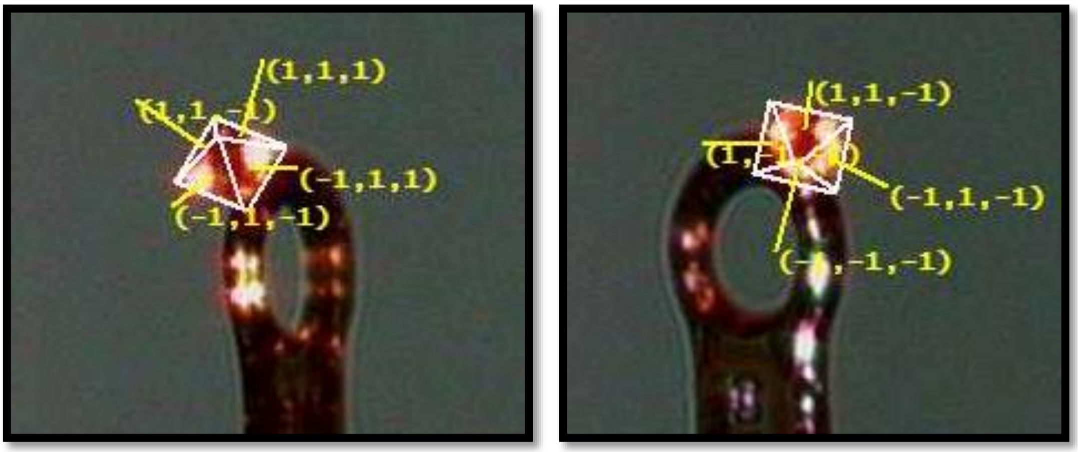

Figure S12: Face-normals of principal faces of the crystal, note the triangular shaped faces (1 1 1), (-1 1 1), (1 -1 1), (1 1 -1), (1 -1 -1), (-1 1 -1), (-1 -1 1) and (-1 -1 -1)

SI-6: Nanoindentation Experiments

The effect of temperature (simulations at 0 K cf experimental at 298 K) was not explored. Despite the higher maximum load which developed upon indentation of UiO-67, the degree of elastic recovery, indicated by the residual depth at P = 0, is broadly similar between the two frameworks. The H values are close to the upper limit for those expected for MOFs.[22]

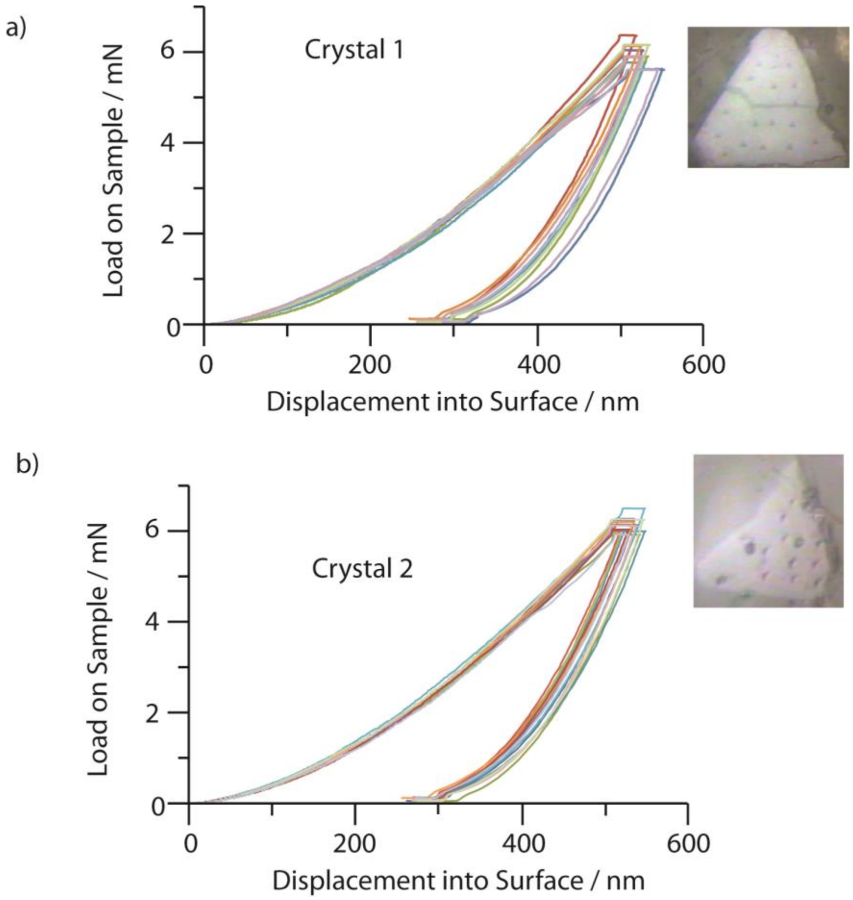

Figure S13: Load-displacement data for the two single crystals of UiO-67 with 17, and 10 indents performed respectively.

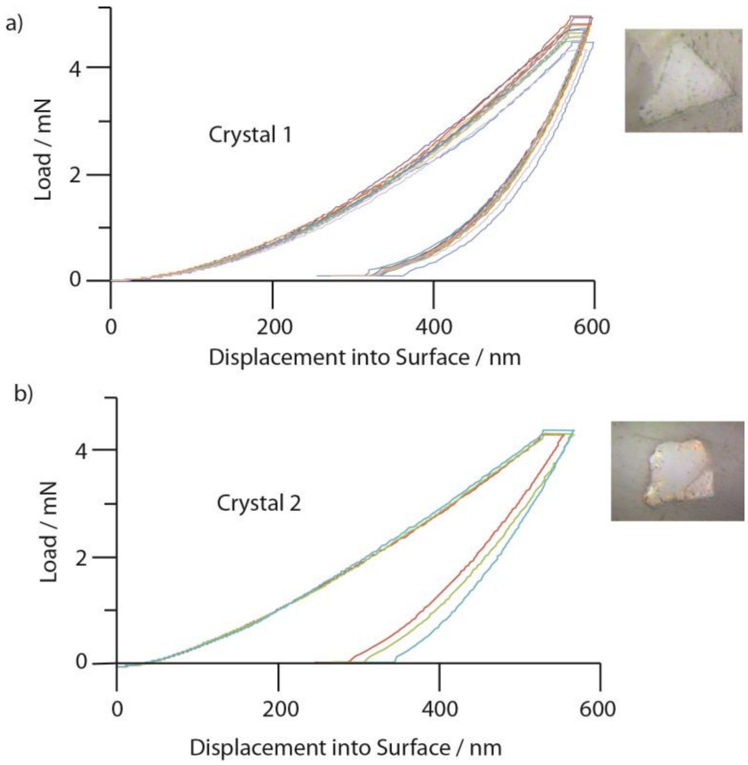

<u>Figure S14:</u> Load-displacement data for the two single crystals of UiO-abdc, with 11, and 4 indents performed respectively.

SI-7: Elastic Moduli

Table S10: Experimental and computational elastic and bulk moduli for UiO-67 and UiO-abdc. All values in GPa.

	K **DFT**[b]	***K*** **DFT** [c]	***K*** **MeOH**[a]	***K*** **FC-70** [a]	***E*** **DFT** [c]	***E*** **exp**[a]	***H*** **exp**[a]
**UiO-67**	25.5	17.4	533	N/A	24.1	20.02	1.27
**UiO-abdc**	16.6	15.2	485	14.8	21.5	13.24	0.65

[a] Experimental values. [b] values from DFT compression. [c] values from elastic calculations

DFT calculations of the elastic stiffness tensors (second-order elastic constants) of both UiO-67 and UiO-abdc were performed at the quantum mechanical level, in the density functional theory approach with localized basis sets (CRYSTAL14 code[23]). We used the PBESOL0 hybrid exchange-correlation functional[24] and all-electron basis sets for all atoms, following the methodology established for both structural and mechanical properties on UiO-66 in refs.[25] Elastic stiffness tensors were calculated and tensorial analysis was performed following the procedure outlined in refs.[26]

The second-order stiffness tensors obtained are (in units of GPa), for UiO-67:

25.78	13.20	13.20			
13.20	25.78	13.20			
13.20	13.20	25.78			
			9.50		
				9.50	
					9.50

and for UiO-abdc:

19.94	11.15	10.99			
11.15	24.17	12.58			
10.99	12.58	23.97			
			9.153		
				7.82	
					8.05

DFT calculations were also performed to characterize the elastic response of UiO-67 and UiO-abdc. The elastic modulus retrieved for UiO-67, *E* = 24.1 GPa, was higher than that for UiO-abdc, *E* = 21.5 GPa (Table 2). Bulk moduli of 17.4 and 15.2 GPa were also extracted from the elastic tensors, which are consistent with the evolution of the unit cell volume in the DFT compression studies at very low pressures (SI-3, Fig. S3).

To confirm the calculated values*,* Young's moduli, *E*, and hardness, *H*, of evacuated single crystals of UiO-67 and UiO-abdc were probed by nanoindentation. Single crystal X-ray diffraction was performed to establish Miller indices of the crystal facets (SI-4). The small size of the crystals (50 µm x 50 µm x 50 µm) necessitated the indents performed on UiO-67 and UiO-abdc to be placed in close proximity of one another, on the (111) facets. The consistency across indents implies that indent proximity was not detrimental to the load-displacement values gained. Similarly, cracking upon polishing did not appear to affect mechanical response of the crystal.

Using the load-displacement data (SI-5) gained during the indentation, *E* and *H* as a function of depth were calculated, indents being performed up to a maximum of 500 nm to prevent further cracking (Figure 6). The average values for each sample were calculated as *E* = 20.02 GPa and *H* = 1.27 GPa (UiO-67), and *E* = 13.24 GPa and *H* = 0.65 GPa (UiO-abdc). Good agreement with DFT values in the case of the former are observed, though results agree less well for the latter. The effect of temperature (simulations at 0 K cf experimental at 298 K) was not explored. Despite the higher maximum load which developed upon indentation of UiO-67, the degree of elastic recovery, indicated by the residual depth at *P* = 0, is broadly similar between the two frameworks. The *H* values are close to the upper limit for those expected for MOFs.[22]

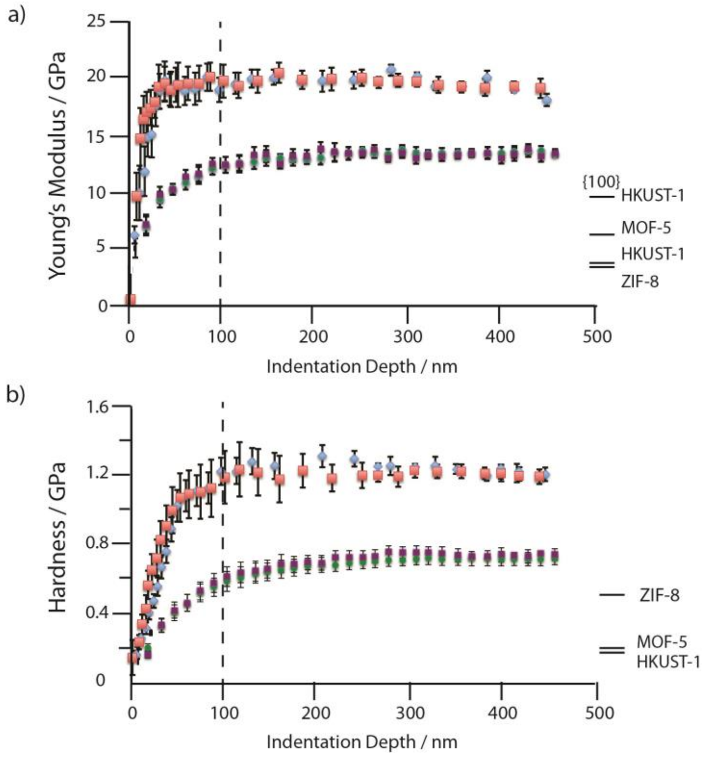

Figure S15: a) Elastic moduli as a function of indentation depth for two single crystal samples of UiO-67 (red and blue), and two single crystals of UiO-67 azobenzene (green and purple). Indented faces were the [111] in each case. Error bars represent the standard deviation of the 10 and 15 measurements performed on crystals of UiO-67, and the 15 and 4 indents performed on crystals of UiO-abdc respectively. b) Hardness values as a function of indentation depth with associated errors as above. In each case, literature values for average values of other MOFs of broadly comparable solvent accessible volume are supplied, alongside a large value for a preferentially oriented film of HKUST-1.[22, 27] Elastic modulus and hardness calculations were performed using data from depths over 100 nm, due to the variance below this level.

The elastic modulus of UiO-67 is amongst the largest reported by nanoindentation for the MOF family, and agrees with the low compressibility of the rigid, planar linker present in the framework. The magnitude of this rigidity is however surprising, given the inversely proportional relationship between *E* and framework solvent accessible volume (SAV). [22] The Mercury software [28] was used to calculate a SAV of 65.9 % for UiO-67 (SI-8), which is moderately high when compared to recent benchmarks in the field.[29] Indeed, HKUST-1 $[Cu_3(C_9O_6H_3)_2]$, a prototypical MOF composed of Cu^{2+} ions linked by benzene-1, 3, 5-tricarboxylate ligands, possesses a SAV of 64.3 %, yet an elastic modulus of just 9.3 GPa [27d].

The framework is also markedly stiffer than the prototypical frameworks ZIF-8 [$Zn(C_3H_3N_2)_2$] and MOF-5 [$Zn_4O(C_8H_4O_4)_3$], of 50.3 % and 77.7 % SAV respectively. [27b, 27c]

The elastic modulus of UiO-abdc is substantially lower than that of UiO-67, which is in agreement with its higher SAV (71.8 %). It is interesting to note that this large decrease in rigidity is accompanied by a relatively small increase in SAV, whereas previous work on the ZIF family of MOFs noted that changes in SAV of *ca*. 20 % would be required to elicit decreases in mechanical response of a similar order (*ca*. 40 %).[27c] This vastly more flexible nature is consistent with our observation of the frustrated, bowed nature of the abdc ligand in UiO-abdc.

SI-8: Solvent Accessible Volume Calculations

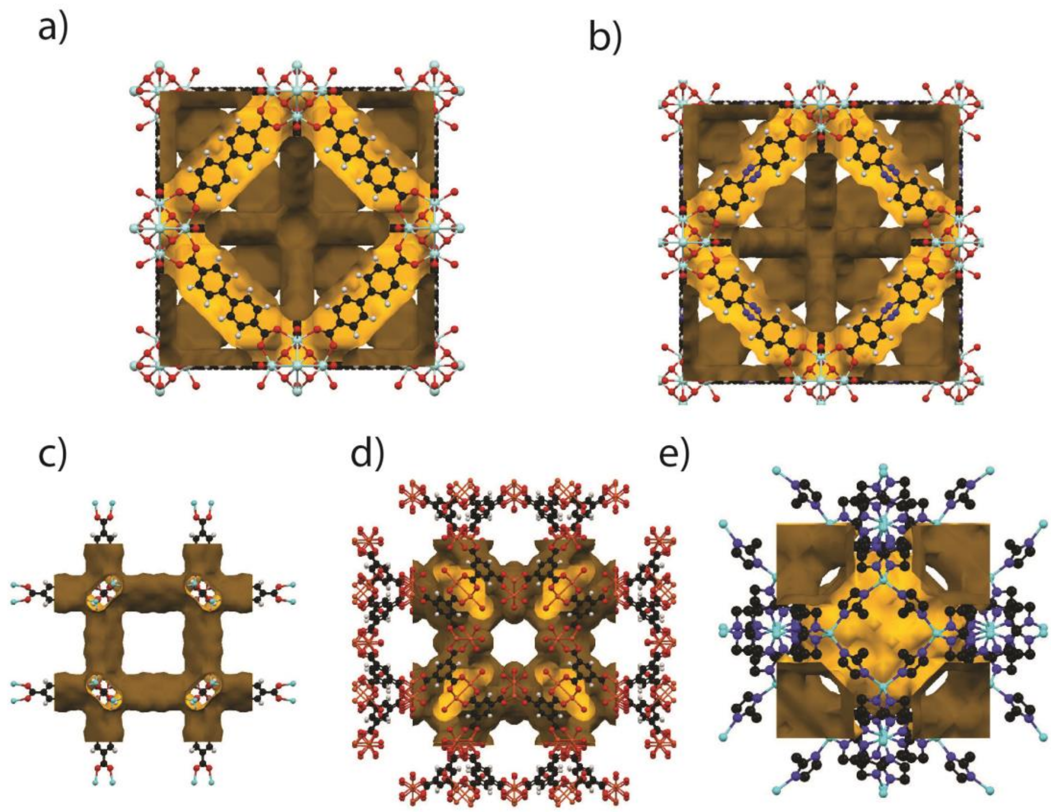

A probe radius of 1.2 Å and grid spacing of 0.7 Å were used.

Figure S16: Solvent accessible volumes for a) UiO-67, b) UiO-abdc, c) MOF-5, d) HKUST-1 and e) ZIF-8.

References:

[1] aB.-A. X. S. Bruker-Nonius, Madison, Wisconsin, USA, **2007**; bG. M. Sheldrick, University of Göttingen, Germany., **2008**.

[2] S. A. Moggach, D. R. Allan, S. Parsons, J. E. Warren, *Journal of Applied Crystallography* **2008**, *41*, 249-251.

[3] G. J. Piermarini, S. Block, J. D. Barnett, R. A. Forman, *J Appl Phys* **1975**, *46*, 2774-2780.

[4] B.-A. X. S. Bruker-Nonius, Madison, Wisconsin, USA, **2006**.

[5] aS. Parsons, **2004**; bB.-A. X. S. Bruker-Nonius, Madison, Wisconsin, USA, **2004**.

[6] P. W. Betteridge, J. R. Carruthers, R. I. Cooper, K. Prout, D. J. Watkin, *Journal of Applied Crystallography* **2003**, *36*, 1487-1487.

[7] L. Palatinus, G. Chapuis, *Journal of Applied Crystallography* **2007**, *40*, 786-790.

[8] A. L. Spek, *Journal of Applied Crystallography* **2003**, *36*, 7-13.

[9] W. C. Oliver, G. M. Pharr, *J Mater Res* **2004**, *19*, 3-20.

[10] J. VandeVondele, M. Krack, F. Mohamed, M. Parrinello, T. Chassaing, J. Hutter, *Comput Phys Commun* **2005**, *167*, 103-128.

[11] aA. D. Becke, *Phys Rev A* **1988**, *38*, 3098-3100; bC. T. Lee, W. T. Yang, R. G. Parr, *Physical Review B* **1988**, *37*, 785-789.

[12] S. Grimme, J. Antony, S. Ehrlich, H. Krieg, *J Chem Phys* **2010**, *132*.

[13] aS. Goedecker, M. Teter, J. Hutter, *Physical Review B* **1996**, *54*, 1703-1710; bC. Hartwigsen, S. Goedecker, J. Hutter, *Physical Review B* **1998**, *58*, 3641-3662; cM. Krack, *Theor Chem Acc* **2005**, *114*, 145-152; dA. L. Benabid, B. Wallace, J. Mitrofanis, C. Xia, B. Piallat, V. Fraix, A. Batir, P. Krack, P. Pollak, F. Berger, *Cr Biol* **2005**, *328*, 177-186; eJ. VandeVondele, J. Hutter, *J Chem Phys* **2007**, *127*.

[14] G. J. Martyna, M. L. Klein, M. Tuckerman, *J Chem Phys* **1992**, *97*, 2635-2643.

[15] aL. J. Chen, J. P. S. Mowat, D. Fairen-Jimenez, C. A. Morrison, S. P. Thompson, P. A. Wright, T. Duren, *Journal of the American Chemical Society* **2013**, *135*, 15763-15773; bM. J. McGrath, J. I. Siepmann, I. F. W. Kuo, C. J. Mundy, J. VandeVondele, J. Hutter, F. Mohamed, M. Krack, *Chemphyschem* **2005**, *6*, 1894-1901; cJ. Schmidt, J. VandeVondele, I. F. W. Kuo, D. Sebastiani, J. I. Siepmann, J. Hutter, C. J. Mundy, *J Phys Chem B* **2009**, *113*, 11959-11964.

[16] aA. M. Reilly, D. A. Wann, C. A. Morrison, D. W. H. Rankin, *Chem Phys Lett* **2007**, *448*, 61-64; bA. M. Reilly, S. Habershon, C. A. Morrison, D. W. H. Rankin, *J Chem Phys* **2010**, *132*; cA. M. Reilly, S. Habershon, C. A. Morrison, D. W. H. Rankin, *J Chem Phys* **2010**, *132*.

[17] I. J. Bruno, J. C. Cole, P. R. Edgington, M. Kessler, C. F. Macrae, P. McCabe, J. Pearson, R. Taylor, *ACTA CRYSTALLOGRAPHICA SECTION B-STRUCTURAL SCIENCE* **2002**, *58*, 389-397.

[18] L. Valenzano, B. Civalleri, S. Chavan, S. Bordiga, M. H. Nilsen, S. Jakobsen, K. P. Lillerud, C. Lamberti, *Chem. Mater.* **2011**, *23*, 1700-1718.

[19] S. J. Clark, M. D. Segall, C. J. Pickard, P. J. Hasnip, M. I. J. Probert, K. Refson, M. C. Payne, *Zeitschrift für Kristallographie* **2005**, *220*, 567-570.

[20] aJ. P. Perdew, K. Burke, M. Ernzerhof, *Physical review letters* **1996**, *77*, 3865-3868; bA. Tkatchenko, M. Scheffler, *Physical review letters* **2009**, *102*.

[21] D. F. Shanno, *Math Comput* **1970**, *24*, 647-&.

[22] J. C. Tan, A. K. Cheetham, *Chemical Society Reviews* **2011**, *40*, 1059-1080.

[23] R. Dovesi, R. Orlando, B. Civalleri, C. Roetti, V. R. Saunders, C. M. Zicovich-Wilson, *Zeitschrift Fur Kristallographie* **2005**, *220*, 571-573.

[24] J. P. Perdew, A. Ruzsinszky, G. I. Csonka, O. A. Vydrov, G. E. Scuseria, L. A. Constantin, X. L. Zhou, K. Burke, *Physical review letters* **2008**, *100*.

[25] aM. J. Cliffe, W. Wan, X. D. Zou, P. A. Chater, A. K. Kleppe, M. G. Tucker, H. Wilhelm, N. P. Funnell, F. X. Coudert, A. L. Goodwin, *Nature Communications* **2014**, *5*; bM. J. Cliffe, J. A. Hill, C. A. Murray, F. X. Coudert, A. L. Goodwin, *Phys Chem Chem Phys* **2015**, *17*, 11586-11592.

[26] aA. U. Ortiz, A. Boutin, A. H. Fuchs, F. X. Coudert, *Physical review letters* **2012**, *109*; bA. U. Ortiz, A. Boutin, A. H. Fuchs, F. X. Coudert, *J Chem Phys* **2013**, *138*.

[27] aB. Van de Voorde, R. Ameloot, I. Stassen, M. Everaert, D. De Vos, J. C. Tan, *Journal of Materials Chemistry C* **2013**, *1*, 7716-7724; bJ. Y. Jung, F. Karadas, S. Zulfiqar, E. Deniz, S. Aparicio, M. Atilhan, C. T. Yavuz, S. M. Han, *Phys Chem Chem Phys* **2013**, *15*, 14319-14327; cT. D. Bennett, J. C. Tan, S. A. Moggach, R. Galvelis, C. Mellot-Draznieks, B. A. Reisner, A. Thirumurugan, D. R. Allan, A. K. Cheetham, *Chem-Eur J* **2010**, *16*, 10684-10690; dS. Bundschuh, O. Kraft, H. K. Arslan, H. Gliemann, P. G. Weidler, C. Woll, *Appl Phys Lett* **2012**, *101*.

[28] C. F. Macrae, I. J. Bruno, J. A. Chisholm, P. R. Edgington, P. McCabe, E. Pidcock, L. Rodriguez-Monge, R. Taylor, J. van de Streek, P. A. Wood, *Journal of Applied Crystallography* **2008**, *41*, 466-470.

[29] M. Bosch, M. Zhang, H. C. Zhou, *Advances in Chemistry* **2014**.
